# Population Genomics Approaches for Genetic Characterization of SARS-CoV-2 Lineages

**DOI:** 10.3389/fmed.2022.826746

**Published:** 2022-02-21

**Authors:** Fatima Mostefai, Isabel Gamache, Arnaud N'Guessan, Justin Pelletier, Jessie Huang, Carmen Lia Murall, Ahmad Pesaranghader, Vanda Gaonac'h-Lovejoy, David J. Hamelin, Raphaël Poujol, Jean-Christophe Grenier, Martin Smith, Etienne Caron, Morgan Craig, Guy Wolf, Smita Krishnaswamy, B. Jesse Shapiro, Julie G. Hussin

**Affiliations:** ^1^Research Centre, Montreal Heart Institute, Montreal, QC, Canada; ^2^Département de Biochimie et Médecine Moléculaire, Université de Montréal, Montreal, QC, Canada; ^3^Department of Microbiology and Immunology, McGill University, Montreal, QC, Canada; ^4^Department of Computer Science, Yale University, New Haven, CT, United States; ^5^Mila – Quebec AI institute, Montreal, QC, Canada; ^6^Research Centre, CHU Sainte-Justine, Montreal, QC, Canada; ^7^Département de Pathologie et Biologie Cellulaire, Université de Montréal, Montreal, QC, Canada; ^8^Département de Mathématiques et Statistique, Université de Montréal, Montreal, QC, Canada; ^9^Department of Genetics, Yale University, New Haven, CT, United States; ^10^Département de Médecine, Université de Montréal, Montreal, QC, Canada

**Keywords:** SARS-CoV-2, population genomics, viral surveillance, haplotype network, variant detection, principal component analysis, lineage annotation

## Abstract

The genome of the Severe Acute Respiratory Syndrome coronavirus 2 (SARS-CoV-2), the pathogen that causes coronavirus disease 2019 (COVID-19), has been sequenced at an unprecedented scale leading to a tremendous amount of viral genome sequencing data. To assist in tracing infection pathways and design preventive strategies, a deep understanding of the viral genetic diversity landscape is needed. We present here a set of genomic surveillance tools from population genetics which can be used to better understand the evolution of this virus in humans. To illustrate the utility of this toolbox, we detail an in depth analysis of the genetic diversity of SARS-CoV-2 in first year of the COVID-19 pandemic. We analyzed 329,854 high-quality consensus sequences published in the GISAID database during the pre-vaccination phase. We demonstrate that, compared to standard phylogenetic approaches, haplotype networks can be computed efficiently on much larger datasets. This approach enables real-time lineage identification, a clear description of the relationship between variants of concern, and efficient detection of recurrent mutations. Furthermore, time series change of Tajima's D by haplotype provides a powerful metric of lineage expansion. Finally, principal component analysis (PCA) highlights key steps in variant emergence and facilitates the visualization of genomic variation in the context of SARS-CoV-2 diversity. The computational framework presented here is simple to implement and insightful for real-time genomic surveillance of SARS-CoV-2 and could be applied to any pathogen that threatens the health of populations of humans and other organisms.

## 1. Introduction

The Severe Acute Respiratory Syndrome coronavirus 2 (SARS-CoV-2) is a highly transmissible virus responsible for the current ongoing pandemic. SARS-CoV-2 is a positive-sense, single stranded RNA genome of 29,903 nucleotides. This virus is transmitted from person to person by droplet transmission. Most SARS-CoV-2 infected patients experience mild to moderate symptoms, such as high body temperature and sometimes respiratory symptoms. However, some patients may experience severe symptoms like pneumonia and acute respiratory distress syndrome. The virus' genome is accumulating mutations at a steady pace since its introduction into human hosts. Genomic surveillance and the identification of variants of concern (VOC), their impact on transmission, disease severity and immune response are of tremendous importance to pandemic control, most notably in the context of worldwide vaccination efforts. In this context, an unparalleled wealth of chronologically and globally sampled viral genomes have been sequenced in a concerted international effort and submitted to public databases such as the Global Initiative for Sharing All Influenza Data (GISAID) (1).

In attempt to track the transmission and spread of emerging lineages, several lineage nomenclatures have been proposed, the most commonly used ones being the clades from Nextstrain and GISAID (phylogenetic approach) and Pangolin annotations (decision tree approach implemented in pangoLEARN) (2). Additionally, the World Health Organization assigns Greek letters to Variants of Concerns (VOCs), Variants Being Monitored (VBM), and Variants Of Interest (VOI). VOCs started emerging at the end of the first year of the pandemic, with the first one reported being the Alpha variant (3). Shortly after that, several other variants have attracted attention from the scientific community and public health bodies, among them Beta, Gamma, and Lambda, and more recently, the Delta and Omicron variants which are becoming globally dominant (4). However, despite these numerous attempts at describing variants, it remains very difficult to find out how these different VOCs are related to each other and to identify quickly from which genomic background they emerged. As all genomic backgrounds may not have the same baseline fitness, this information is of importance for efficient viral diversity surveillance.

Historically, phylogenetics is used to describe relationships between viral sequences. However, it is becoming more computationally intensive to use with the increasing number of closely related sequences made available on GISAID. Specifically, very low diversity between sequences can sometimes only be explained by sequencing uncertainty, potentially leading to falsely resolved monophyletic groups. Phylogenetic reconstructions can also fall into local minima because of weak phylogenetic signal due to rugged likelihood surface (5). Furthermore, as the SARS-CoV-2 data continue to accumulate in real-time from multiple sources during this global pandemic, it is not possible to keep the phylogenetic trees up to date to track thousands of sequences a day. Thus, there is a clear need to update our computational analysis pipelines to resolve the bioinformatics bottleneck problem (6). Alternatively, by taking advantage of established population genetic paradigms that study the evolution of mutation frequencies in time within sequences that are closely related, we may be able to describe and analyze the increasingly large SARS-CoV-2 datasets. For instance, an early study constructed a haplotype network to visualize circulating lineages of the SARS-CoV-2 virus (7). Importantly, while phylogenetic approaches assume that the ancestral sequences are unobserved and represented by internal nodes, a haplotype network approach is appropriate when internal nodes actually are observed, because some sampled sequences are ancestral to others. This is the case for the current sampling scheme of pandemic sequences worldwide, despite many sampling biases (8–10). Currently, effective reproductive number (R) is a widely used metric to define outbreaks and to measure COVID19 disease spread (11). Tajima's D (12), a classical population genetics approach, can also be used to investigate SARS-CoV-2 lineage expansions (13). Dimensionality reduction techniques summarizing genetic diversity are also widely employed to investigate population structure in various species, and in particular, principal component analysis (PCA) has been proposed to investigate the population structure of SARS-CoV-2 virus early in the pandemic (14).

Here, we use genomic data collected from GISAID (1) during the fist year of the pandemic (January to December 2020) to present a computational genomic pipeline for large-scale viral genetic profiling using a collection of established population genetic approaches. Using these tools, we aim to characterize the full scale of genetic diversity during the pre-vaccination phase of the pandemic, making use of all available GISAID sequences. We highlight the limitations of alternative methods and provide access to this pipeline, which can be easily applied to the data generated in subsequent years of the pandemic. We show that these methods are useful to characterize the evolutionary steps undertaken by the virus during its early adaptation to human hosts. This computational framework can help design efficient preventive strategies, identify potentially expanding, divergent lineages and derive a fast response against viral adaptation to future therapeutic strategies.

## 2. Results

### 2.1. Viral Genetic Data Pre-processing

**Figure 1:**
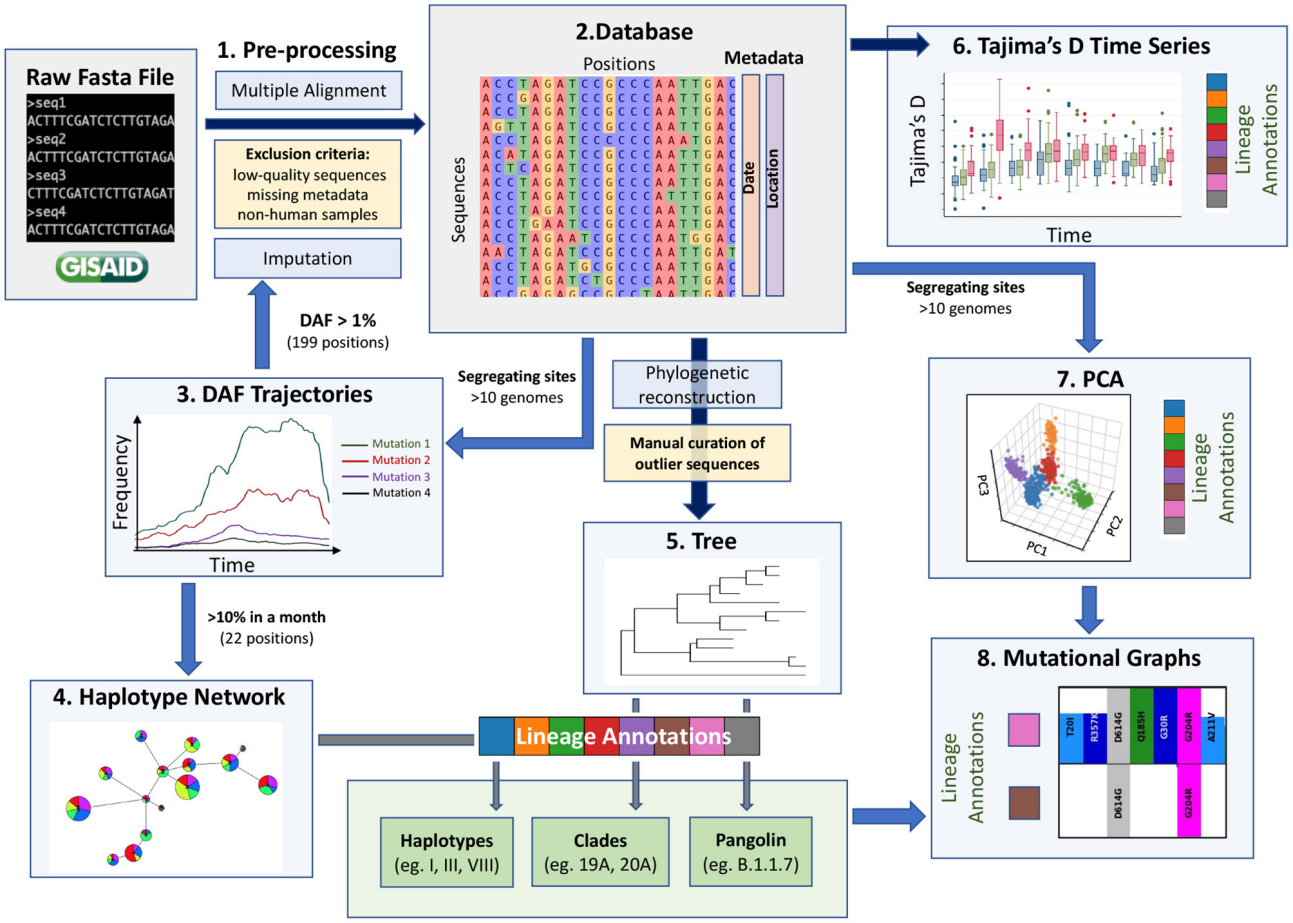
A data-driven methodological pipeline for analyzing viral genomic data. This workflow recapitulates the major analysis steps used to analyze SARS-CoV-2 consensus sequence data submitted to GISAID during the first year of the COVID-19 pandemic. Dark blue arrows represent steps where all positions are kept (except spurious sites), blue arrows represent steps where subsets of positions are kept (indicated next to the arrow), yellow boxes represent filtering steps at the level of sequences, light blue boxes represent the methodological steps and the main steps are numbered from 1 to 8. These population genetic and unsupervised learning methods constitute a comprehensive toolbox to allow the scientific community to monitor the evolution of the virus efficiently. Box plot modified from Bejarano (15).

A key challenge in extracting meaningful information from genomic data is careful pre-processing to exclude low quality sequences, artifacts associated with diverse sequencing technologies and missing data. The GISAID database has stringent submission guidelines and quality checks that guarantee a minimum quality of the data. However, the diversity of submitting institutions and heterogeneity of submission time points leads to a heterogeneous pool of sequences. We thus recommend adding pre-processing steps (Step 1, Figure 1) to obtain a more homogeneous dataset and remove as many technical biases as possible. Specifically, our pipeline flags a series of systematic errors induced by sequencing and bioinformatic methodologies (Methods), which were more common in the first months of the pandemic. From the raw fasta file with 384,407 consensus sequences downloaded from GISAID on January 19th 2021, we obtained a high-quality dataset of 329,854 consensus sequences. We aligned these high-quality sequences and extracted all RNA substitutions compared to the reference sequence (NC_045512.2) (16).

We note that the sequencing effort across the world has been heavily biased, as we have 4,194 sequences from Africa, 23,499 sequences from Asia, 210,624 from Europe, 72,774 from North America, 15,009 from Oceania and 3,735 sequences from South America. These numbers do not reflect the case counts in each continent but the resources available to track and sequence SARS-CoV-2 genomes. This bias can be detrimental when it comes to understanding the virus's evolution and lineage tracing.

Currently, missing data within consensus sequences, reflected as N characters in the sequences, is a main source of variant misassignment. To help reduce this missing data problem and improve variant assignment, we next imputed all sequences using ImputeCoVNet (17) at positions where the derived allele frequency (DAF) was over 1% during at least one month (199 positions). This novel approach uses a 2D residual neural-network autoencoder that has an accuracy of >99% and surpasses distance-based methods in terms of computation time (17), which is a major advantage for such a large dataset. Given the very low level of recombination that has been reported so far, imputation of prevalent mutations is very accurate for SARS-CoV-2 genomic sequences, such that this step greatly benefits downstream analyses. We then built a harmonized database of RNA substitutions (Step 2, Figure 1) that contains a total of 24,802 mutated genomic positions (Supplementary Table S1).

**Figure 2:**
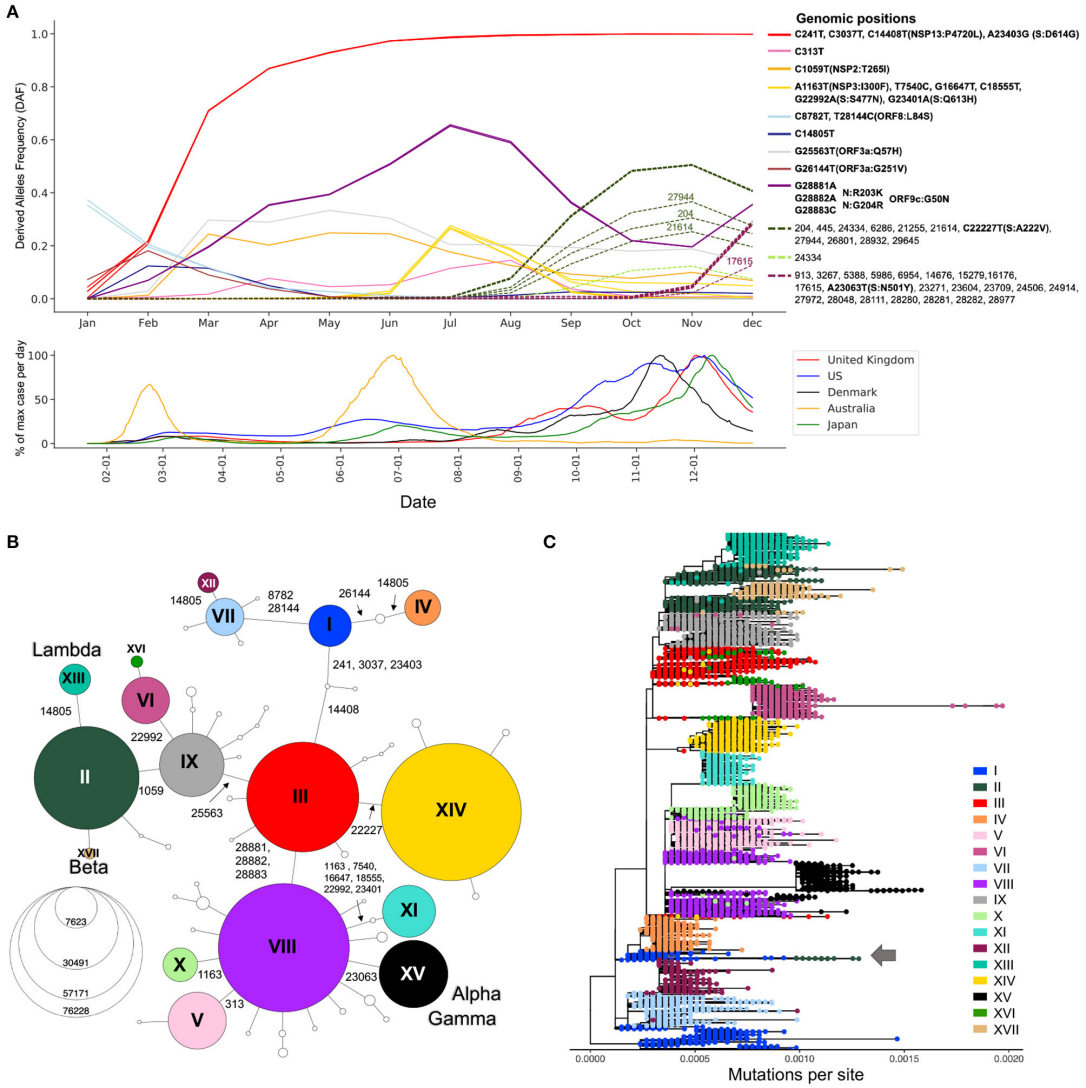
Viral genetic diversity during the first year of the pandemic. **(A)** Top panel shows Derived Allele Frequencies (DAF) over time of representative high-frequency substitutions during the first year of the pandemic. Only positions that exceed a DAF of 10% for a given month are shown. Positions with highly correlated DAF trajectories (*r*^2^>0.99) have the same line color. Solid lines are used for mutations appearing in the first wave of the pandemic (January–July), and dotted lines show mutations appearing during the second wave of the pandemic (August–December). Bottom panel shows the daily case counts in the top five countries from which we have the most GISAID sequences. The *y*-axis represents the % of maximum cases per day rolled over 14 days. On the *x*-axis, only ticks of the first of the month are represented. **(B)** Haplotype network representing genetic subtypes based on representative mutations (position underlined in A). Genomic positions that differ between two nodes (haplotypes) are specified on edges. Nodes are colored by haplotype and node size represents the number of consensus sequences for each haplotype. The 17 main haplotypes are annotated with roman numerals. **(C)** Divergence tree made from 15,690 SARS-CoV-2 consensus sequences using FastTree using a GTR+Gamma20 model and TreeTime to refine the divergence tree. The haplotype network built from prevalent mutations using all high-quality consensus sequences recapitulates the phylogeny well.

Since most of the consensus sequences in this dataset are from the UK (43%) and the USA (20%), we define two waves in the first year of the pandemic, corresponding to the two successive global increases of COVID-19 cases observed in countries that implemented strict containment measures in March 2020 and then relaxed these measures in the summer of 2020, with the caveat that countries from the southern hemisphere had offset waves due to seasonality (Figure 2A, bottom) (18). The first wave comprises sequences sampled from January to the end of July 2020, whilst the second wave comprises those sampled from August to December 2020 (19). We identified 20,403 substitutions within the first wave consensus sequences and 22,210 in the second wave, relative to the reference genome (Supplementary Table S1). Each of these mutated positions were further categorized based on their frequency of occurrence in the global host population during the first and second waves of the pandemic (Supplementary Table S1). Singletons make up 23 and 17% of the first and second wave mutations, respectively. Because these mutations are only seen in one sequence in each wave, they are most likely enriched in sequencing errors. Doubletons (mutations seen twice during a wave) account for 14% (first wave) and 11% (second wave) of the mutations, consistent with an expanding population. In any given wave, most of the mutations impacting viral fitness will be found in more than 100 sequences (20). In our data, such mutations made up 5 and 11% of the first and second waves, respectively.

### 2.2. Derived Allele Frequency Trajectories Through Time

Expanding viral lineages will harbor a set of prevalent genetic mutations that quickly increase in frequency over the course of an epidemic. To detect these mutations during the first year of the COVID-19 pandemic, we considered their derived allele frequencies (DAF) over time (Step 3, Figure 1). In the first wave, 20 RNA substitutions reached a DAF of 10% for at least one month (January to July) (Figure 2A). Four mutated positions are in linkage disequilibrium with each other, as evidenced by their overlapping DAF trajectories, meaning that they co-occurred together. Three of these substitutions are C-to-U mutations (C241U, C3037U, C4408U), while the last one is A23403G in the Spike protein (S:D614G). These four substitutions increased very quickly in frequency (Figure 2A, red DAF trajectory). This lineage was the first to become dominant and did so in a few months, climbing to 71% in March (Figure 2A). It is thought that S:D614G is the driver of this event and has been shown to have a selective advantage for SARS-CoV-2 which is conferred by an increase in transmission and viral load in the respiratory tract (21). Three consecutive co-occurring substitutions G28881A, G28882A, and G28883C also increased in frequency during the first wave (Figure 2A, purple DAF trajectory). It is the only consecutive tri-nucleotide change, or triplet, that reached a DAF over 1% in the first year of the pandemic, an event that is unlikely to arise by chance and could represent an adaptive change occurring on a codon. These three mutations span two amino acids in the N protein, leading to N:R203K and N:G204R. However, in the overlapping gene ORF9c (or ORF14) (22) they form a single codon mutated from GGG to AAC, causing a single missense change in the resulting protein (ORF9c:G50N). Interestingly, ORF9c is a novel gene in SARS-CoV-2 compared to known human coronaviruses (23), coding for a putative transmembrane protein. Additionally, this codon change substantially disrupts the RNA secondary structure of this specific region of the SARS-CoV-2 genome, destabilizing a local *Y*-shaped structure into a “wobbly” loop by increasing its free energy 11.2% and its sub-optimal base-pairing diversity by 24.5% (Supplementary Figure S1). Finally, the first wave was marked by the increase in frequency of a group of six co-occurring substitutions: A1163T, T7540C, G16647T, C18555T, G22992A, and G23401A (Figure 2A, yellow DAF trajectory). This lineage peaked in July 2020 and was mainly circulating in Australia (24).

The second wave is marked by an increase in the number of prevalent mutations compared to the first wave: from August to December, 33 new high-frequency substitutions arose (Figure 2A). The DAF trajectories for these mutations show two well-defined groups, representing two different lineages: one with mutation S:A222V at genomic position 22,227 (Figure 2A, dashed green DAF trajectories) and a lineage with mutation S:N501Y at position 23063 (Figure 2A, dashed plum DAF trajectories). The lineage with S:A222V corresponds to 20E in Nextstrain annotations (G in GISAID Clade annotation) and was first reported by Rambault and colleagues (25, 26). This lineage accumulated 11 co-occurring mutations during its expansion and was mostly seen in European countries. The lineage with S:501Y mainly corresponds to the Nextstrain 20I, now commonly known as the Alpha variant, which accumulated 22 co-occurring high frequency mutations during its expansion (Figure 2A) (25–27).

### 2.3. Haplotype Networks for Fast Evolutionary Clustering of Sequences

The generation of haplotype networks is a widely used approach for analyzing and visualizing the relationships between sequences within a population (28, 29). The nodes of the network are haplotypes, edges represent mutated genetic positions that vary between two nodes (Figure 2B). The size of the nodes generally varies to represent the number of sequences for a specific haplotype. In the case where there is little to no recombination, this approach results in a minimum spanning tree. To keep the number of nodes tractable for informative visualization, we defined the haplotypes by selecting the 22 mutations displayed in the DAF trajectories that are most representative of the virus's genetic diversity during the first year of the pandemic (Methods, Figure 2A underlined positions, Supplementary Figure S2). We generated a haplotype network (Step 4, Figure 1) using a technique that takes the time of sampling into account (Methods). We included the 122 haplotypes with more than 10 sequences in our representation, ignoring rare events. The final haplotype network includes 17 main haplotypes (Figure 2B), representing the main genetic lineages circulating during the pandemic's first year and several “descendant” haplotypes. Haplotype I includes all sequences with ancestral states at each position (i.e., reference haplotype) and haplotype XV corresponds mainly to the Alpha variant, differing from the reference haplotype at 8 positions. The haplotype network representation also helps clarify the relationships between specific VOCs, with both Beta and Lambda emerging as sublineages on a haplotype II genomic background, whereas Alpha and Gamma arise as sublineages on a haplotype VIII genomic background.

**Figure 3:**
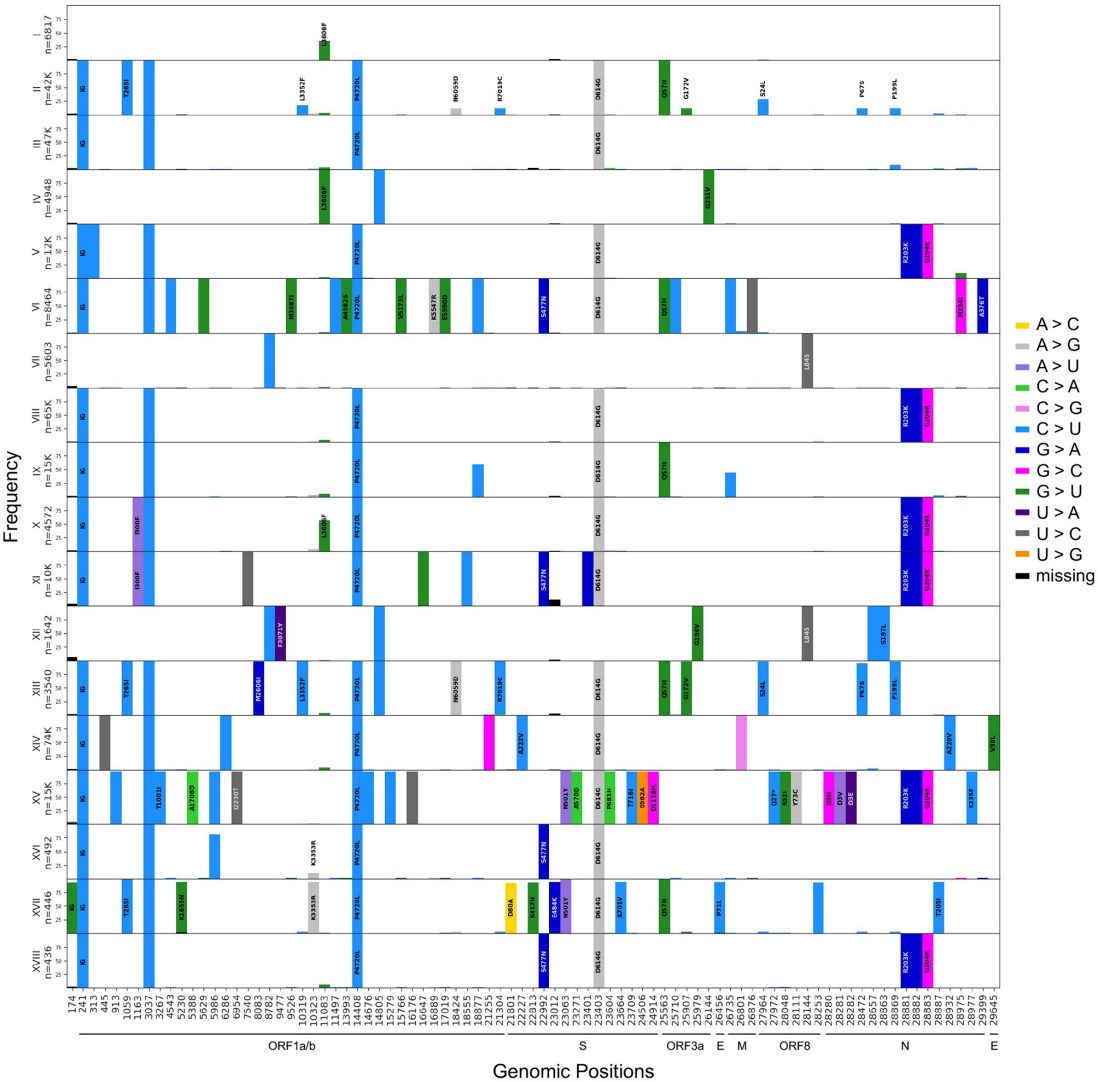
Mutational signatures of the 17 major haplotypes. Aligned histograms of each of the main 17 haplotype groups. The *y*-axis of each histogram represents the frequency within each haplotype of mutations that differ from the reference nucleotide in at least 90% of the sequences represented. On the *x*-axis, each bar represents a mutated position colored by its substitution type and is labeled with the corresponding amino acid change (no labels are displayed for synonymous mutations). The annotation was done using SnpEFF (30).

For the main haplotypes (I to XVII), we created mutational graphs that represent the mutational landscape of each subgroup of sequences (Figure 3). These graphs are stratified histograms of DAFs for genomic positions that differ from the reference sequence in at least one haplotype. The visualization highlights a mutational signature unique to each haplotype. The mutational jumps are also made obvious by this representation, where we see a large number of lineage-specific mutations for haplotypes VI and XV, in addition to XVII (Beta) in the Spike protein. This representation also allows the detection of homoplasy, for example at position 11,083, which is fixed in haplotype IV (V GISAID Clade) but is also seen at various frequencies in multiple other haplotypes.

**Figure 4:**
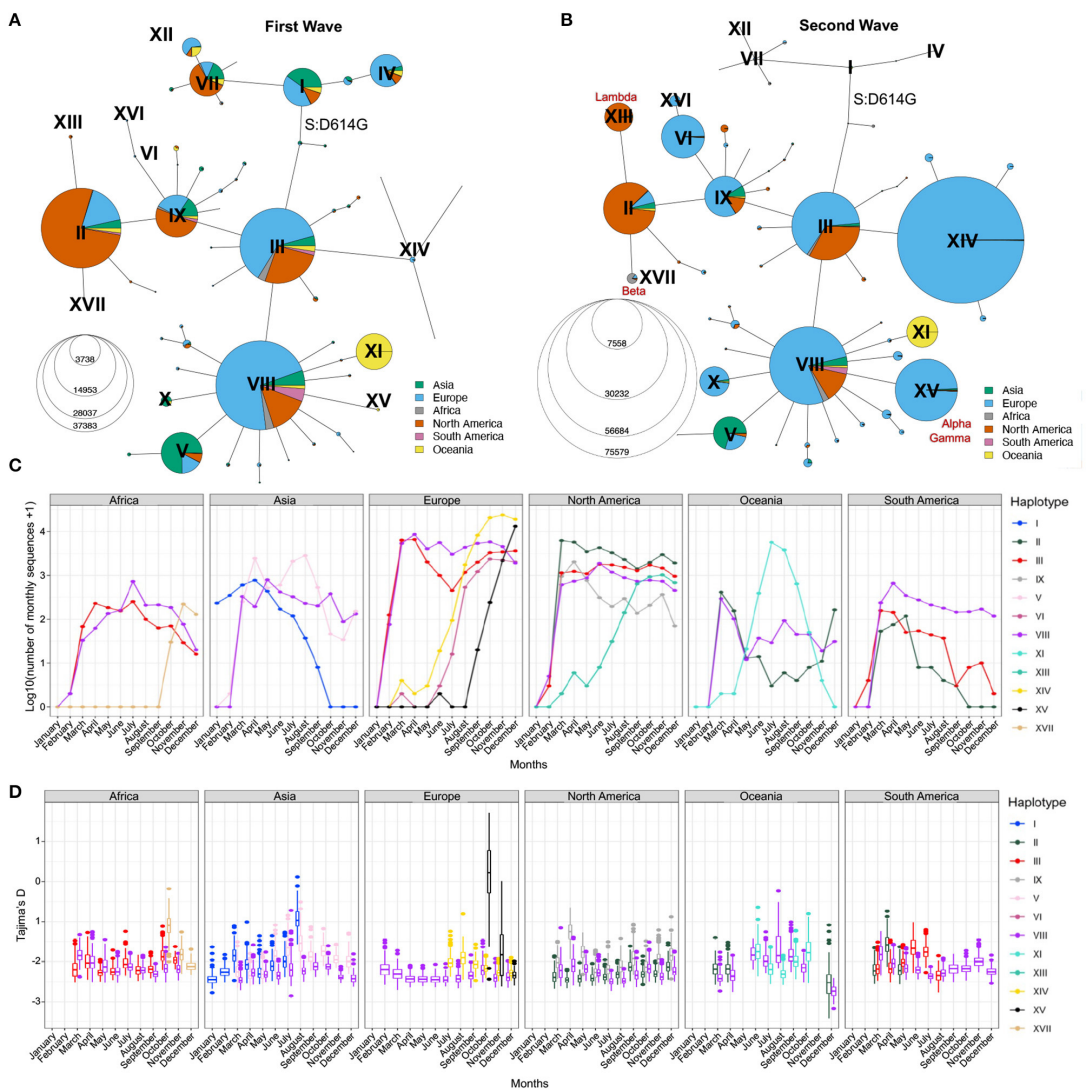
Distribution of SARS-CoV-2 sequences in space and time. Haplotype network of the first **(A)** and second **(B)** waves. Node size represents the number of consensus sequences for each haplotype and pie charts represent continental proportions for each haplotype. **(C)** GISAID consensus sequence counts (on a log10 scale) of the most prevalent haplotypes on each continent during the first year of the pandemic. **(D)** Tajima's D estimates of the three most prevalent haplotypes on each continent for the first year of the pandemic. Box plots represent 500 estimates of Tajima's D from random resamplings of 20 genome sequences for each month with at least 20 sequences. Both the haplotype network and Tajima's D are insightful tools for detecting expanding lineages at a given point in time.

To further visualize the genetic diversity of the virus and the relationships between viral sequences in the first two waves, we looked at the viral diversity during each wave of the pandemic using the haplotype network (Figures 4A,B). During the first wave, the haplotype network shows the presence of nine haplotypes with more than 200,000 sequences (II–V, VII–IX, XI, XII) diverging from the ancestral haplotype I (Figure 4A), with haplotypes II, III, and VIII being the most prevalent, all carrying the S:D614G mutation. Most haplotypes are seen in several regions of the world, albeit at different frequencies (e.g., V arose mainly in Asia and II mainly in North America), except for haplotype XI. This latter lineage (Pangolin D.2) was mostly circulating in Australia, forming 92.8% of all high-quality Australian sequences from GISAID in July 2020. First detected in June 2020, this variant almost completely vanished as of October 2020. The second wave is marked by a critical decrease in the prevalence of haplotypes without the S:D614G mutation (I, IV, VII, and XII, almost becoming extinct by August 2020) and the fast increase in prevalence of haplotypes VI, XIV, and XV, which arose mainly in Europe. Other novel region-specific haplotypes (XIII in North America, XVI in Europe, and XVII in Africa) arose during this wave (Figure 4C). Additionally, visualizing root-to-tip distances (Supplementary Text, Supplementary Figure S4B), we observed that the second wave was marked by the appearance of haplotypes with mutational jumps, the most apparent ones resulting in haplotypes VI and XV.

### 2.4. Comparison to Phylogenetic Reconstruction

To compare the haplotype network to a more standard phylogenetic approach (Step 5, Figure 1), we generated a divergence tree using FastTree and TreeTime along with other complementary tools (see Methods), as recommended in multiple published pipelines (31, 32). Additionally, to conform with what is done in the literature, the phylogenetic tree was constructed without an outgroup of a distant lineage, and we considered the ancestral lineage as the outgroup. With these approaches, using all 329,854 sequences is computationally intensive, representing a bottleneck that can prevent fast real time surveillance, therefore, most strategies use sub-sampling of datasets (Methods) which can bias the representation of the circulating diversity of the viral population. In contrast, the haplotype network can be built with all available sequences, while recapitulating the phylogenetic structure quite accurately, despite some lineage splitting, particularly of haplotype I (Figure 2C). The phylogenetic approach sometimes wrongly combines very distant lineages, for example lineage I and II (Figure 2C, arrow) where further inspection of these sequences puts into doubt the relationship reported by the phylogenetic tree.

**Figure 5:**
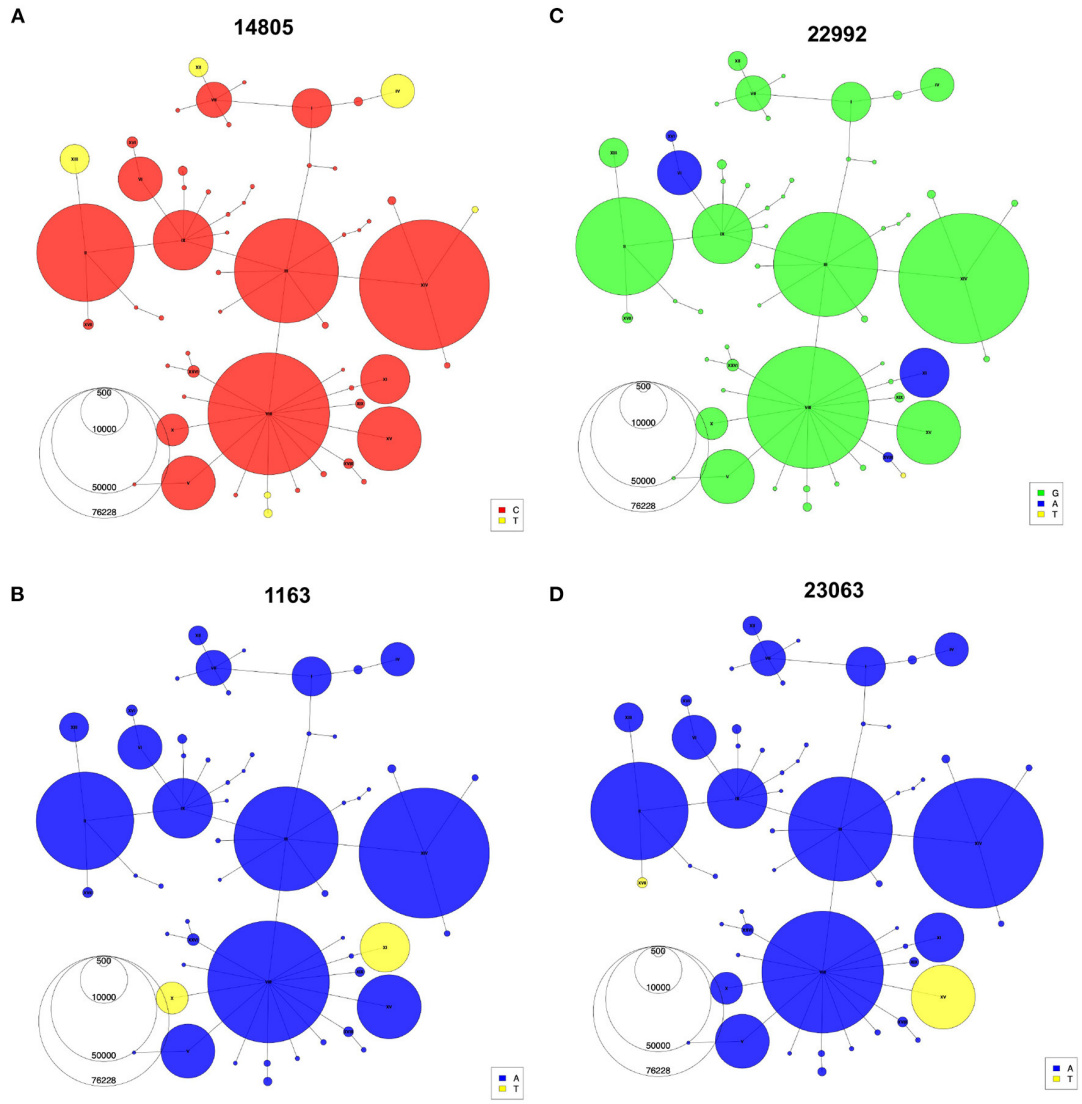
Recurrent mutations visualized using the haplotype network. Haplotype Networks colored according to the presence of specific alleles at genomic positions 14,805 **(A)**, 1,163 **(B)**, 22,992 **(C)**, and 23,063 **(D)**. Node size represents the number of the first year of the pandemic consensus sequences for each haplotype.

Indeed, with the haplotype network annotations, we are able to identify problematic connections between sequences in the phylogeny that would otherwise go undiscovered. Once identified, these false connections can then be corrected by additional tools and manual adjustments (Supplementary Text, Supplementary Figure S3). Additionally, the haplotype network allows easy representation of recurrent mutations that occur independently multiple times on several genomic backgrounds. For instance, the C-to-U mutation at position 14805 (Figure 5A) emerged on three different backgrounds (IV, XII, XIII). Haplotypes X and XI share a mutation at position 1163 in ORF1a/b (Figure 5B), but each is part of an independent mutational jump. Sequences from haplotype XI include the Spike missense mutation S:S477N (genomic position 22992), which is a recurrent mutation that also defines haplotype VI (Figure 5C), a distantly related lineage that expanded in Europe during the second wave (33) (Figure 4C). Finally, the mutation at 23063 (S:N501Y), which defines haplotypes XV and XVII (Figure 4D), has appeared multiple times since the emergence of SARS-CoV-2 (34). Nevertheless, phylogenetic and molecular-clock analyses can bring complementary information to the haplotype network, for instance, by allowing estimation of the time to a most recent common ancestor (TMRCA) and mutation rate. After thinning using Gblocks (35) to remove unresolved parts of the alignment (see Methods), the TMRCA was estimated to be in October 2019 and the mutational rate estimate was 21.60 mutations/year, in line with Nextstrain and other estimates (36, 37). However, alternative data pre-processing steps and parameters choices led to different values (see Supplementary Text), highlighting again that these phylogenetic results should be interpreted with caution.

### 2.5. Comparison to Other Lineage Annotation Systems

Our haplotype definition is well in line with Nextstrain's lineage definition and GISAID clade annotation system (Supplementary Figure S2A), though some distinctions exist. For instance, the haplotype approach can differentiate sequences from haplotype I and IV, differing by two substitutions (genomic positions 26144,14805), and from III and IX, differing at position 25583, whereas Nextstrain does not differentiate these lineages, grouping them into 19A and 20A, respectively. Similarly, GISAID clade annotation groups sequences from haplotypes III and XIV into clade G despite differing by a substitution (genomic position 22227). We note that these three examples show disagreement between GISAID and Nextrain annotations, and basing a nomenclature system on haplotype annotations can reconcile the two. In contrast, our categorization of sequences by haplotype and the Pangolin annotation methodology (3) do not agree well (Supplementary Figure S2B) with, for instance, the B.1 lineage spanning many genetically distant haplotypes: sequences from haplotype II and VIII can be assigned to the same generic B.1, while these differ by a total of five high frequency mutations, including the triplet at 28881-28883. Conversely, lineages B.1.1, B.1.2, and B.1.5 are sub-lineages of haplotypes VIII, II, and III, respectively, defined by mutations that never surpassed 10% worldwide. These inconsistencies between the genetic background of different Pangolin lineages and the greater granularity observed compared to Nextstrain lineages justify the usage of haplotype categories for the analyses of genetic evolution of SARS-CoV-2. However, the haplotype definition based only on these 22 most prevalent mutations in 2020 does not differentiate all VOCs. For example, the sequences of the Gamma variant discovered in Brazil (38), which also has the substitution at position 23063 (S:N501Y) as well as the triplet, are grouped with Alpha sequences in haplotype XV.

### 2.6. Time Series Change of Tajima's D Statistic to Detect Lineage Expansions in Real Time

The haplotype network representation informs on the overall size of clusters but lacks information on the lineages' expansions over time, especially by geographical region. Population genetic statistics, most notably Tajima's D, has been used to estimate epidemiological parameters of pandemic influenza A (H1N1) (39) and can detect population expansion and contraction events. Specifically, an excess of low-frequency alleles in the population results in a strongly negative Tajima's D value, indicating a rapid population expansion. However, Tajima's D is very sensitive to population structure and is, therefore, not meaningful when applied on a global scale. However, the genetically-informed grouping of sequences by haplotypes as well as stratification by world regions provides the opportunity to get closer to specific viral populations, where Tajima's D can be applied. We computed Tajima's D in each month of 2020 for the three most prevalent haplotypes in each continent (Step 6, Figure 1) while controlling for sample size (Section Methods). We recognize that the uneven sequencing coverage across the continents may bias mutation rate estimates, therefore we avoided comparing regions. We then correlated Tajima's D to the number of sequences per haplotype per continent and observed a moderate negative correlation (mean adjusted R^2^ = 0.24, s.d. = 0.23, Supplementary Figure S5A). This correlation indicates that Tajima's D time series can recapitulate the major variations in the number of sequences per haplotype per month across each continent (Figures 4C,D). Indeed, we observe a decrease of Tajima's D through time for several haplotypes that either took over in a specific region or are known to have become dominant (Figures 4C,D; Supplementary Figures S5B,C). For instance, in Africa, haplotype XVII (Beta) appears in October 2020 with a high Tajima's D value compared to dominant haplotypes VIII and III, and then shows a fast decrease of Tajima's D in the next months, consistent with population expansion (Figure 4D). This correlates with the drastic increase in the number of sequences seen at the end of 2020 (Figure 4C), reflecting the emergence of Beta in South Africa (40). Another striking event of this type is seen in Europe where the rapid increase of XV (Alpha) coincides with a steep decrease in Tajima's D from October to December (Figures 4C,D; Supplementary Figure S5B). These events are also consistent with observations from Singh and Yi (41) who tracked the spread of the corresponding Nextstrain clades (XV: Nextstrain 20I; XVII: Nextstrain 20H).

In North America, Tajima's D for haplotype IX also shows a marked decrease from April to June, suggesting an expansion of this lineage, although its prevalence in the sampled population from GISAID did not increase (Figure 4C). This may reflect undersampling of specific populations in North America. Conversely, the rapid rise of haplotype XIII (Lambda) is captured both by Tajima's D and sequence counts (Supplementary Figure S5C). Other types of events can be seen, such as loss of lineages and lineages causing outbreaks that are quickly contained. An example of the former is seen in Asia where SARS-CoV-2 emerged (42), for which Tajima's D values of haplotype I sequences (GISAID S clade) increase with time, reflecting the drop in diversity and a contraction of population size of the ancestral lineage, which became almost entirely extinct across the globe, with the last seven sequences sampled in Asia in September 2020. The signature of a contained outbreak is seen in Oceania, where the distribution of Tajima's D across time is U-shaped, indicating an increase in population size followed by a contraction, in line with sequence counts (Figure 4C). In South America, sample counts per haplotype suggest that haplotype VIII out-competed II and III by the end of 2020, but the low Tajima's D values in August 2020 are inconsistent with a decrease of these lineages and suggest that the number of sequences assigned to these haplotypes is an underestimate in that region.

### 2.7. Fine-Scale Viral Population Structure Using Principal Component Analyses

**Figure 6:**
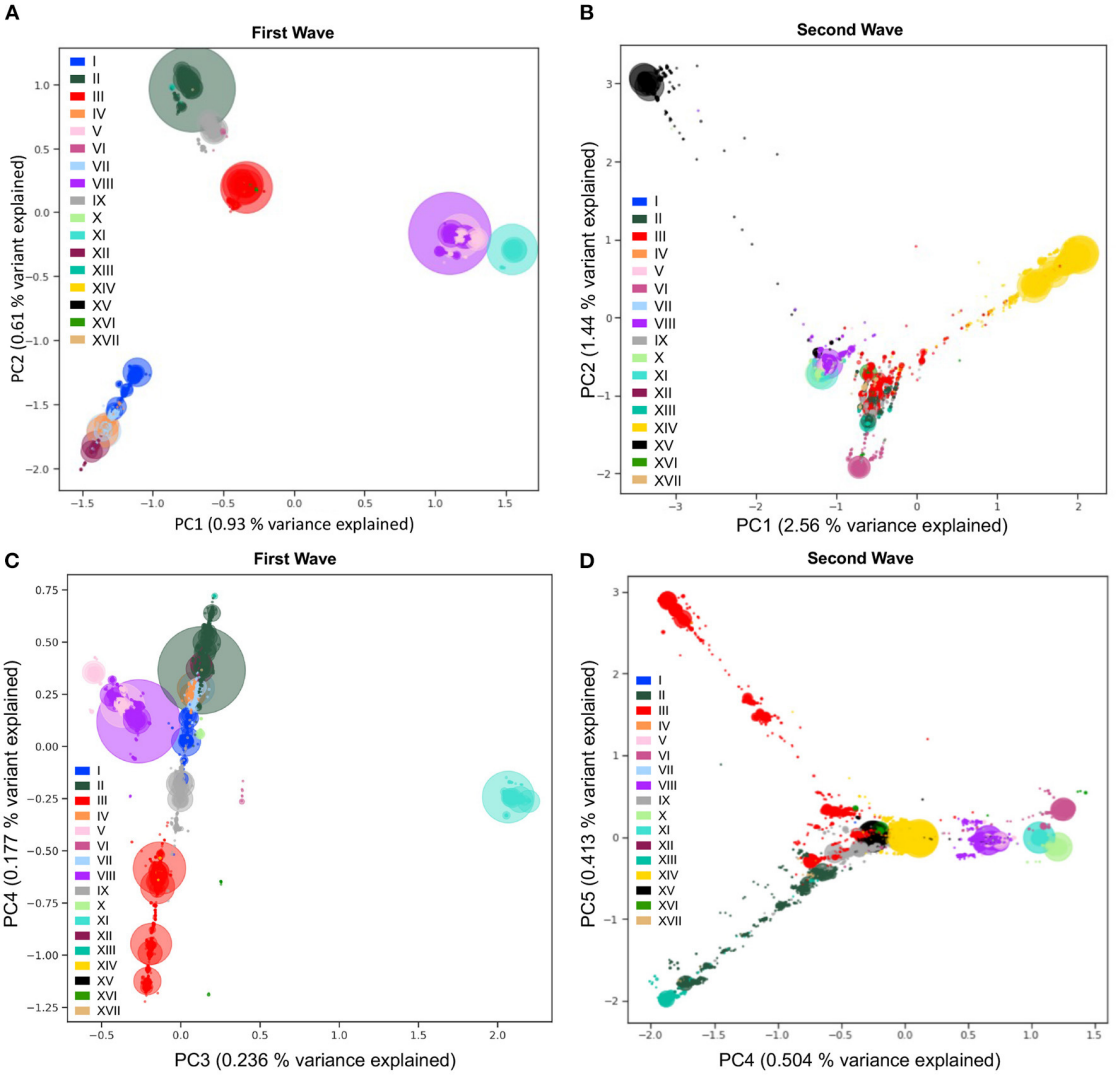
Viral population structure during the first and second waves of the pandemic. Principal Component Analyses (PCA) of genetic diversity of the first **(A,C)** and second **(B,D)** waves' consensus sequences reveal the population structure of the 17 main haplotypes in each wave. Genetic variation present in at least 10 genomes is used. The PCA is computed with all sequences, and only the sequences from the 17 main haplotypes are projected. Identical sequences are projected onto the same coordinates, therefore, the number of sequences represented by each point is proportional to the size of the dots, with added transparency. PC1 and PC2 show differentiation between the main lineages from the two waves **(A,B)**. The variant responsible for the Australian outbreak stands out clearly on PC3 from the first wave **(C)** and the Lambda variant sequences (XIII) are shown as the most distal subgroup on PC4 and PC5 from the second wave **(D)**, in opposition to sequences from haplotype VI (on PC4) and subgroups of haplotype III (on PC5). The PCA recapitulates insightful characteristics of the evolutionary relationships of sequences and identifies major lineages from the two pandemic waves.

To detect and visualize fine-scale structure in genetic variation, a common statistical approach is to use PCA of coded alleles at mutated positions segregating in populations. The projection of sequences onto the principal components is known to reflect the underlying (generally unknown) genealogical relationships between haploid sequences (43). In the viral populations of SARS-CoV-2 from 2020, we performed PCA (Step 7, Figure 1) on viral mutations present in at least 10 sequences from the first and second waves (Figure 6). The first two PCs describe the most variation between sequences and clearly show discrimination between haplotypes dominating during either of the two waves (Figures 6A,B). The coordinates of clusters relative to one another in the first wave agree with the haplotype network representation (Figure 4A): on PC1, the haplotypes without S:D614G are separated away from VIII and XI carrying the triplet mutations, whereas PC2 separates them from haplotypes II and IX, with haplotype III located at the centre, in line with its intermediate position in the haplotype network. Additionally, the distance in the PC1/2 space between haplotype groups appears to recapitulate very well the genetic distance between them. For instance, haplotype III sequences are at least four mutations away from I, three mutations away from VIII, and two mutations away from II, reflecting distances between groups on the graph (Figure 6A). In the second wave, PC1 and PC2 do not reflect these phylogenetic relationships as much, but rather highlight the most divergent groups (Figure 6B). Both PCs show the XV group as an extreme group, which is explained by the major mutational jump of 22 mutations from the haplotype VIII background defining Alpha. We can, however, see a subset of XV sequences clustering with VIII sequences, which are either precursor sequences of Alpha, or Gamma sequences. Beyond PC1 and PC2, other PCs from the two waves show additional structure within and between haplotype subgroups (Supplementary Figure S6). For instance, PC3/4 of the first wave sequences show the divergence of haplotype XI (Figure 6C), the lineage dominating in Australia in the summer, whereas PC4/5 of the second wave sequences show the emergence of the haplotype XIII lineage (Beta) from its ancestral background on haplotype II (Figure 6D). Inspecting additional PCs allowed us to detect the emergence of subgroups in specific haplotypes (Supplementary Figure S6), with, for example, a diverging group in haplotype VII (also known as GISAID L lineage and Nextstrain 19B lineage) which seemed to have accumulated additional mutations compared to other sequences in this lineage early on (PC13 and PC14 in Supplementary Figure S6A), which could represent an early mutational jump that did not spread widely.

### 2.8. In Depth Exploration Into Lineages of Interest

**Figure 7:**
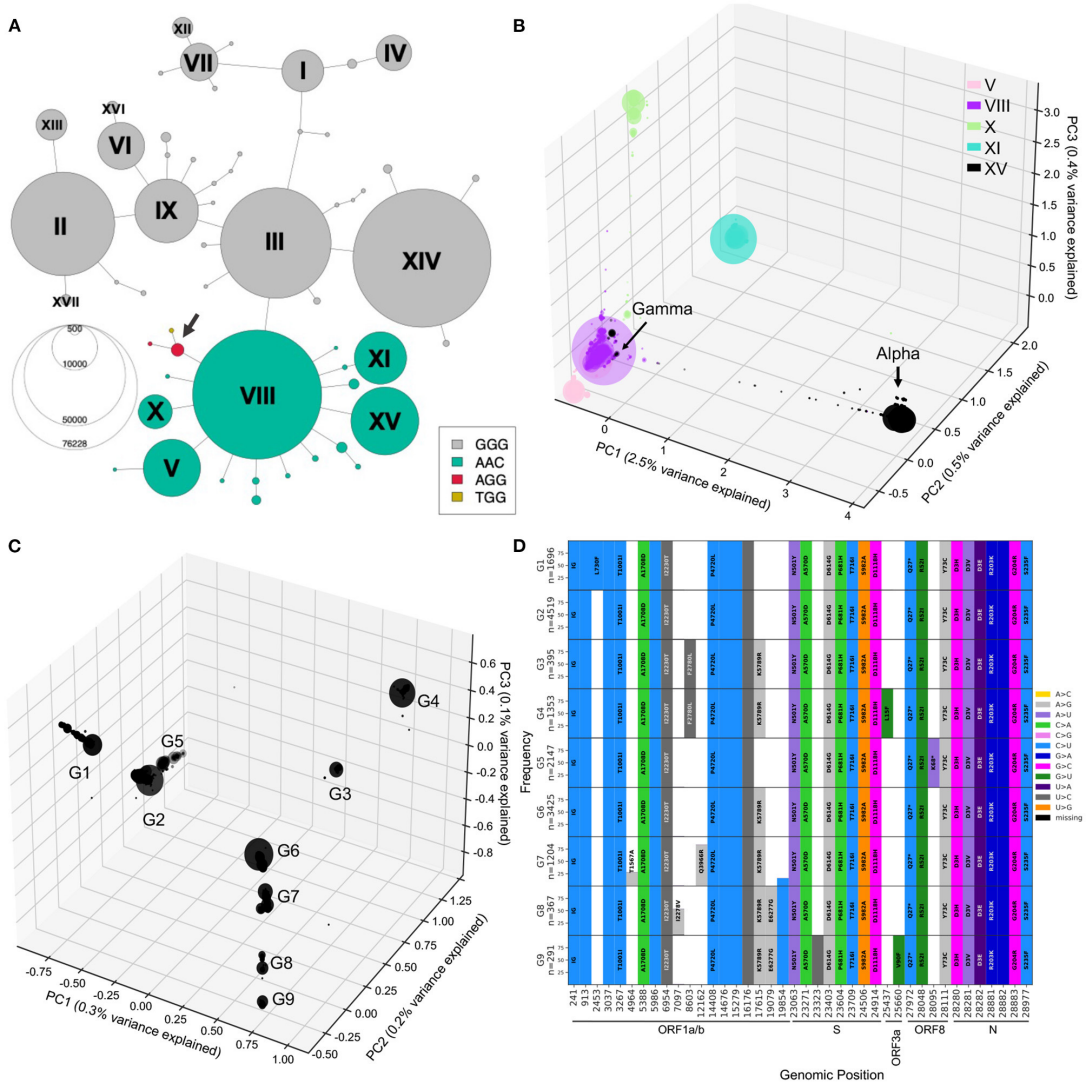
Mutational landscape of haplotype VIII and its descendant lineages. **(A)** Haplotype network colored according to the alleles at positions 28881-28883. Two additional low-frequency combinations had emerged at this locus with genotypes AGG and TGG (arrow). **(B)** PCA generated from sequences from haplotype VIII and descendants V, X, XI, and XV. PC1, PC2, and PC3 explaining 3.5% of the variation were plotted into three axis. **(C)** PCA visualization of 0.6% of the variation within Alpha annotated sequences, PC1, PC2, and PC3 plotted onto three axis. PC1/2/3 reveal 9 major groups, arbitrarily labeled G1 to G9. **(D)** Mutational graphs reporting mutations seen in at least 25% of the sequences in each group in C. Bars are colored by substitution type, and the corresponding amino acid changes are shown, as in Figure 3. Genomic position annotation was done using SnpEFF (30).

With the previous tools, we identified different genetic lineages of SARS-CoV-2 that were predominant in the first year. We can also investigate descendant lineages, that arose from main lineages and increased faster than the parental lineages over time. To illustrate this, we chose to focus on the subset of the viral sequence space that have mutations within the 28881-28883 triplet (Figure 7A). Since the appearance of haplotype VIII (first sequence sampled February 16, 2020), Alpha and Gamma have emerged on this genetic background, and in 2021, Omicron, the three of which include the S:N501Y mutation. We selected all sequences from the five distinct main haplotypes with the ACC nucleotide triplet combination (V, VIII, X, XI, and XV) and performed a PCA of genetic diversity in these sequences (Figure 7B; Supplementary Figure S7). PC1, PC2, and PC3 reflect the phylogenetic relationships between haplotype VIII and its descendent haplotypes, with sequences from haplotypes V, X, XI, and XV forming distinct groups around haplotype VIII sequences (Figure 7B). Similar to the second wave PCA, PC1 is mainly explained by the set of mutations that appeared on haplotype VIII to generate the Alpha variant (2.5% of variance explained) and allows clear distinction between Alpha and the Gamma lineages. PC2 and PC3 allows us to visually separate the other haplotypes, with PC2 explained by divergence between V and XI and PC3 separating sequences from haplotype X. Furthermore, PC4 to PC20 show several subgroups within the defined haplotypes; for instance PC4 splits the V group into two distinct sub-lineages, and PC19 shows distinct groups of XI sequences, while the other PCs seem to reflect the genetic heterogeneity within VIII sequences (Figure 7B; Supplementary Figure S7).

To investigate further Alpha lineages, we performed a PCA only on sequences annotated Alpha (B.1.1.7) by Pangolin (Figure 7C, Supplementary Figure S8), which corresponds to haplotype XV. The three-dimensional PCA plot of Alpha annotated sequences shows additional structure within that lineage, with nine main groups arising (G1-9). To understand the mutational landscape of each group of Alpha/XV sequences, we generated mutational graphs specific to these groups (Figure 7C).

The G2 group has no additional mutations to the Alpha-defining mutations, suggesting that this group is the ancestral lineage. It is separated, on PC1, from other groups of sequences with a mutation at genomic position 17615, in ORF1ab (G3,4,6-9). The G5 group shows a nonsense mutation at genomic position 28095 (ORF8:K68*). Interestingly, ORF8:K68* increased in frequency in the first months of 2021 as Alpha was spreading, and was found in over 80% of Alpha sequences by September 2021, revealing a potentially beneficial mutation on the Alpha background (Supplementary Figure S9).

## 3. Discussion

The worldwide efforts to sequence and share thousands of viral genome sequences made in depth tracking of SARS-CoV-2 evolution possible over time, as it spread across the world. However, processing, analysis, and interpretation of hundred-thousands of sequences and mutation events is a challenging task (44). Here, we first proposed a pre-processing pipeline to improve downstream analysis by ensuring high-quality data and imputing missing alleles at key positions to facilitate annotation and therefore lineage tracing. Indeed, after imputation, we were able to recover sequences that would have otherwise been excluded in the haplotype network, and therefore miss key intermediate events (key mutational events). Detection of spurious sites due to sequencing errors and biases is also an important step, and data analysts should be careful about these when processing large amounts of genomic data, especially in a small genome of 29Kb, where by now, every position in the genome has been affected by sequencing errors.

We used population genetic approaches to explore and identify emerging SARS-CoV-2 lineages. Allele frequency tracing through time is a widely used population genetic approach that can monitor circulating lineages over time. Overall, SARS-CoV-2 experienced a period of relative evolutionary stasis during its first months infecting human hosts, consistent with other reports (20, 45). Mutations providing increased viral fitness started emerging and led to a number of VOCs. Indeed, the second wave of the pandemic was marked by the appearance of lineages with mutational jumps which may reflect adaptive steps in the evolutionary trajectories of SARS-CoV-2 within the human host, with the virus acquiring selective advantages to the host immune system (46–49). We observe that the virus has experienced mutational jumps that increased in size (i.e., the number of mutations within a jump) over time, with S:D614G occurring alongside 3 other mutations during the first wave, S:N501Y occurring along side 21 other Alpha mutations during the second wave, and more recently, the new VOC Omicron has acquired a total of 50 mutations on haplotype VIII, which include S:N501Y and S:S477N.

The nomenclature has become increasingly complex with the number of lineages and sub-lineages emerging throughout the world. For the Pangolin or Nextstrain naming, it is unclear how different lineages are from each other, and, therefore, it is not obvious to know on which genomic background a lineage is occurring. However, knowing about the past history of a specific lineage is very important to understand and potentially predict its evolution and impact. Some lineages could evolve specific properties, such as hypermutability or loss of a gene (e.g., ORF8 knock-down, as for Alpha G5 sequences), which could help in understanding their epidemiological impact. We explored tracing lineages using a haplotype network that was generated using the most frequent mutational events in a given time period in order to clarify the genetic background of VOCs in 2020. Interestingly, the Delta variant that emerged early 2021 arose on haplotype III background, but was accompanied with one of the triplet mutations, G to A at position 28881 (N:R203M). One current limitation of the haplotype network presented here is that it is based on a fixed set of mutations, that may not be the ones that are relevant in the next waves. It is however easy to accommodate new mutations from successive waves, as new haplotypes can be generated with any mutational events of interest on any region of interest, for instance the Spike protein, the main vaccines target currently.

Using Tajima's D, a classical neutrality test statistic, we captured the expansion and decline of major circulating haplotype populations in each continent, and correlate them to sequence counts in specific regions of the world. Inconsistencies between Tajima's D predicted expansions and decreases in sequence counts could be an indication of undersampling in a given region, which is a limitation. Sampling biases are numerous in this dataset, and attempts to correct them may also lead to other systematic errors. Furthermore, it is a very unbalanced dataset in terms of sampling countries, with 144,376 (44%) of the sequencing effort during the first year done in the UK. Global strategies aiming at a more uniformly distributed sequencing effort between countries would enable the identification of early emerging variants. Overall, correlating Tajima's D values with epidemiological data at finer geographical scale might improve its explanatory power and inform public health agencies about the epidemiological trends. Essentially, Tajima's D can be used in combination with effective reproductive numbers (R) to estimate the spread of an expanding population in a given region (13). More recently a Genomic Identity (GENI) score (a genome diversity metric) was formulated from SARS-CoV-2 genomic data to estimate outbreak trends that lead to the emergence of new variants (50). This score increases when a population expands and could also be used in a similar way as Tajima's D, in a haplotype-specific manner to account for viral population structure and genetic background.

Population structure during the first year of the pandemic was successfully visualized using PCA in each wave. PCA can thus reveal insightful characteristics of the viral genetic data, and has the potential to identify growing lineages, but grouping of data points (here, sequences) in PCA derived from genetic variation is known to be heavily influenced by uneven sampling of sequences, which means that the number of sequences sampled within sub-lineages will influence distances between subgroups (43). This limitation can be problematic for early detection of new differentiated sub-lineages, which are often sampled in lower numbers compared to the other well-established lineages. Using PCA on a subset of sequences from a specific lineage (e.g., haplotype VIII, Figure 7) revealed fine-scale structure and highlighted diverging groups, defined by specific mutations. However, a limitation of this is that proper clustering using PCA is not always obvious and the field would benefit from novel hierarchical methods that provide real-time clustering of sequences according to their genomic relationships to predict emerging variants.

We presented here a series of population genetics-based analyses to ease lineage tracing of SARS-CoV-2 variants and understand the evolutionary relationship between emerging ones. During the first year of the pandemic, there was no clear evidence of recombination events occurring, as also demonstrated by our analyses. However, because these population genetic approaches are developed and tested with the assumption that the viral sequences are non-recombining, it can constitute a limitation in application if such events start emerging. New reports supporting evidence of recombination events have started emerging, including evidence of recombination between B.1.1.7 and B.1.177 lineages and evidence supporting the recombining origins of lineage B.1.628 (51). More recently, a group reported the first case of an intra-host recombination event during a co-infection with Delta and Gamma of the sample in the study (52). This is the first reporting of a recombination event during co-infection of an individual. The higher rate of potential co-infections, as more transmissible variants sweep the nations, will increase the chance of fitter recombinants lineages arising. Fortunately, the haplotype network approach is an appropriate tool to detect potential recombination of dominant haplotypes, which will look like cycles in the network, and future work will adapt the network reconstruction to accommodate such departure from the minimum spanning tree representation we currently use.

In conclusion, these approaches constitute a comprehensive toolbox to allow the scientific community to continually and closely monitor the evolution of any viral population. In particular, we found that population genetics tools such as haplotype networks, Tajima's D, and PCA give a more detailed genetic diversity analysis of SARS-CoV-2 than existing surveillance strategies only based on phylogenetic trees. In the case of the ongoing pandemic caused by the SARS-CoV-2 virus, developing a dynamic, global and up-to-date understanding of viral evolutionary strategies will be of utmost importance to rapidly respond to emerging variants, identify increasingly infectious or vaccine-escaping lineages, and locate at-risk populations.

## 4. Methods

### 4.1. Pre-processing Details

We downloaded a total of 384,407 sequences from GISAID on January 19th 2021. We then removed samples from non-human hosts as well as those with incomplete sequences (<29, 000bp), for a total of 339,427 sequences. Each consensus sequence was mapped separately to the SARS-CoV-2 reference genome (NC_045512.2) using minimap2 2.17-r974 (53). All mapped sequences were then merged back with all others in a single alignment bam file. The variant calling was done using bcftools mpileup v1.9. https://samtools.github.io/bcftools/ in haploid calling mode. Sequences were processed by batches of 1,000 sequences to overcome technical issues in processing of very low frequency variants within a bam file. Once the variant calling was obtained for each batch, INDELS were removed and bcftools merge was used to merge all the variant calls across the entire dataset. Variants located in both ends of the genome, which have high levels of missingness (>20%, positions 1-54 and 29838-29903) were excluded. We then flagged spurious variants within these sequences (see below) and identified 361 samples with at least two flagged positions, which we removed from our dataset. Of the remaining 339,066 sequences, we excluded sequences without GISAID metadata and with incomplete sampling date (sampling month unavailable), which resulted in a final dataset of 329,854 high-quality consensus sequences. We divided this dataset into two pandemic waves: 139,515 sequences with a submission date between January 1st and July 31st 2020 were defined as first wave samples, and 190,339 sequences with a submission date between August 1st and December 31st 2020 were defined as second wave samples. A mutation database was built using the sqlite3 library. Only positions that are variant from the reference, including missing calls, were included in the *position* table of the SQL database. For phylogenetic analyses, the multiple sequence alignment of 360,026 consensus sequences from 2020 provided by GISAID was downloaded on May 12th 2021.

### 4.2. Spurious Sites Flagging

Positions that were masked by (54) were removed. We additionally developed a tool to flag spurious variants within consensus sequences due to sequence misalignment in the original labs, which we initially detected by inspecting consensus sequences manually These errors were found in larger proportions in the sequences uploaded to GISAID in the early stages of the pandemic. Our approach identified substitutions compared to the reference genome that were located within 10 genomic positions of stretches of N, defined as at least 5 consecutive Ns. This strategy was applied to the 339,427 consensus sequences from human host, we identified 2,164 sequences with at least one flagged position (0.6%). Among these, flagged positions where the mutated allele otherwise reached 1% in one of the pandemic waves (for a total of 199 positions) were considered real mutations and not as spurious sites. A total of 6,736 spurious sites were detected and the variant allele was replaced by N in the sequences. Furthermore, we removed 361 samples with at least two flags. Additional details on this procedure can be found in Supplementary Text. The code is available here https://github.com/HussinLab/covid19_mostefai2021_paper.

### 4.3. Imputation

For the 199 positions reaching 1% derived allele frequency (DAF) in the consensus sequences of one of the two pandemic waves, we imputed the missing alleles using ImputeCoVNet (17). Briefly, ImputeCoVNet is a 2D convolutional ResNet autoencoder that aims to learn and reconstruct SARS-CoV-2 sequences with the help of two sub-networks: (1) an encoder that is responsible for embedding the given input into a low-dimensional vector, and (2) a decoder that is responsible for reconstructing that sequence from that low-dimensional vector. During training, the encoder network takes as input complete sequences encoded with a one-hot representation and the decoder outputs a reconstructed version. Once trained, the model was used to infer missing values within incomplete sequences: the missing alleles at previously defined positions of interest were predicted by the model during reconstruction. We evaluated imputation accuracy on sequences without missing data and reached an accuracy of 99.12% on this set of mutated positions. The code is available here https://github.com/HussinLab/covid19_mostefai2021_paper.

### 4.4. Identification of High-Frequency Representative Substitutions

The 329,854 high-quality consensus sequences were binned into months according to collection date. Monthly DAF for each substitution (24,802) was computed using consensus sequences available for each month. A total of 53 substitutions with DAF over 10% in at least one month were considered to be high-frequency substitutions, 20 in the first wave and 33 in the second wave. In the second wave, the DAF trajectories (i.e., DAF per month for each mutation) were highly correlated (Pearson *r*^2^>0.99), forming two distinct groups of substitutions: for each of the groups, only one mutation was retained, genomic positions 22,227 and 23,063. With the 20 high-frequency substitutions from the first wave, a total of 22 high-frequency substitutions were considered to be representative of the evolutionary trajectories of SARS-CoV-2 in 2020 (Supplementary Table S2).

### 4.5. Haplotype Network

The 22 representative positions were used to define viral haplotypes, which consisted of a group of alleles at these positions that are inherited together from a single parental sequence. We obtained a total of 463 unique haplotypes, and only those with more than 50 sequences were kept, for a total of 56 haplotypes. A haplotype network representing distance relationships between haplotypes was built. Because SARS-CoV-2 sequences were sampled sequentially through time, the haplotype network takes the temporal dimension into account. We split the year into 24 intervals representing half-months and each consensus sequence was attributed to one time interval. For each time interval, a haplotype network was generated using the haplonet function of the pegas R package (55) by including only sequences that occurred before or within this time interval. The networks were merged iteratively over time. At each step, if the merging created a cycle (i.e., the addition of a haplotype that is linked with two previously linked haplotypes) we removed the branches of the cycle that link the haplotypes for which the first occurrence was the longest timeframe. If many links had the longest timeframe, we removed the link between the more differentiated haplotypes. This process solved several time inconsistencies. The code is available here https://github.com/HussinLab/covid19_mostefai2021_paper.

### 4.6. Phylogenetic Analysis and Molecular Clock

To reduce the dataset to allow feasible phylogenetic analyses, we applied several filters: (1) we kept only sequences from the 17 main haplotypes and identical consensus sequences were merged, keeping the earliest collection date as the annotation; (2) outlier sequences in terms of their number of mutations at a given date were excluded; and (3) we sampled at least 3 samples per date per haplotype and then balanced the sampling up to a maximum of 1000 samples per haplotype. The resulting dataset had a total of 15,690 sequences. The sites identified as problematic for phylogenetic tree reconstruction (problematic sites list v. 2021-04-15) were removed (54). The phylogenetic tree was computed using FastTree v2.1.11 (56), an approximately-maximum-likelihood method, using a GTR + Gamma20 model. The divergence tree obtained was then refined using TreeTime, a molecular-clock phylogeny inference method (v. 0.7.4) (57) and was trimmed for excessive long branches using TreeShrink (58). The root-to-tip distance was computed using TempEst v1.5.3 (59) and tree visualization was made using ggtree (60). To compute mutation rate and TMRCA, we used a refined alignment obtained using Gblocks thinning method (35) with default parameters, prior to applying the pipeline described above. All code used in this study is available at https://github.com/HussinLab/covid19_mostefai2021_paper.

### 4.7. Tajima's D

To track SARS-CoV-2 haplotypes' spread, we used a population genetic metric that can infer changes in effective population sizes by comparing the contribution of low- and intermediate-frequency mutations to viral genomic diversity, i.e., Tajima's D (12). We calculated Tajima's D at the continent level to be able to relate its time series to the haplotype network. For each haplotype and each of the 12 months of the first and second waves, we randomly sampled 20 viral consensus sequences from each continent to calculate Tajima's D, and repeated this procedure 500 times to obtain confidence intervals. Lineages or time bins with fewer than 20 sequences were discarded. This sub-sampling method allowed us to control for differences in sample size across continents and time, although sampling biases inevitably resulted in reduced detected diversity. After calculating Tajima's D, we correlated it to the number of sequences per haplotype per continent per month, which we used as a proxy for the number of cases per haplotype per continent per month. We also performed this correlation for each haplotype separately. We evaluated the significance of the correlation using the permutational ANOVA (n=5000 permutations) implemented in the R package “lmPerm” (v.2.1.0) (61). These analyses were implemented in R and are available on Github (https://github.com/arnaud00013/SARS_CoV_2_haplotypes_Tajima_D_2020_time_series/).

### 4.8. Dimensionality Reduction Techniques

Principal Component Analysis (PCA) was performed on the first and the second waves high-quality consensus sequences. Genomic positions with more than 10% of missing samples were removed from analysis: 247 for the first wave and 6 for the second. We kept only derived alleles at a position when seen in at least 10 samples, which resulted in a final set of 6,163 mutated positions for the first wave and 9,818 for the second one. Triallelic and quadriallelic sites were coded as separate mutations. Missing data is encoded as reference allele. We used the incremental PCA method (62).

## Data Availability Statement

Publicly available datasets were analyzed in this study. This data can be found at: https://www.gisaid.org, GISAID.

## Code Availability Statement

The code is available here: https://github.com/HussinLab/covid19_mostefai2021_paper, the data is hosted on GISAID (1).

## Author Contributions

FM: substantial conception, data pre-processing, data analyses, figure conception, drafting of the article, and revising the final version of the paper. IG, AN'G, JP, AP, and VG-L: data analyses, drafting of methods sections, and revising the final version of the paper. JH, CLM, and DJH: analysis conception, figure conception, and revising the final version of the paper. RP and J-CG: data pre-processing, data analyses, figure conception, drafting of methods sections, and revising the article for final approval. MS, EC, MC, GW, and SK: supervision of data analyses and revising the final version of the paper. BJS: conception, supervision of analyses, and revising the article for final approval of the submitted paper. JGH: substantial conception, supervision of analyses, figure conception, drafting of the article, and revising the final version of the paper.

## Funding

This study was supported by funding from the Fonds de recherche du Qubec Sant (FRQS), Canada Foundation for Innovation, IVADO COVID-19 Rapid Response grant (CVD19-030), the Montreal Heart Institute Foundation, the National Sciences and Engineering Research Council (NSERC), Alliance COVID-19 Grant (#ALLRP 554923-20), the Canadian Institutes of Health Research (CIHR) (#174924), and National Science Foundation (NSF: #1636933 and #1920920). MC, EC, and JGH are FRQS Junior 1 Research Scholars and GW holds a Canada CIFAR AI chair.

## Conflict of Interest

The authors declare that the research was conducted in the absence of any commercial or financial relationships that could be construed as a potential conflict of interest.

## Publisher's Note

All claims expressed in this article are solely those of the authors and do not necessarily represent those of their affiliated organizations, or those of the publisher, the editors and the reviewers. Any product that may be evaluated in this article, or claim that may be made by its manufacturer, is not guaranteed or endorsed by the publisher.
