# Supplementary material for: Population Genomics Approaches for Genetic Characterization of SARS-CoV-2 Lineages
Source: Front Med (Lausanne). 2022 Feb 21;9:826746. doi: 10.3389/fmed.2022.826746 (PMC8899026; doi:10.3389/fmed.2022.826746)
Supplement: Supplementary file 1 [file Data_Sheet_1.pdf]

Supplementary Material

Supplementary Text

Additional Details on Phylogenetic Analyses

A multiple sequence alignment of 360,026 consensus sequences provided by GISAID was downloaded (version of January 20th 2021, downloaded on May 12th 2021). Sequences from the 17 most frequent haplotypes were kept for further analysis. To avoid over-representation, identical consensus sequences were merged and represented by the first one seen (earliest date). Only GISAID sequences with a date between January and December 2020 were kept. Samples diverging too much in regard to their sampling date were then removed following this equation $j \times 0.0597 + 50$ where j is the number of days since the January 1st 2020. This means that we allowed the mutation rate to be a bit above one mutation every 16 days. The factor 0.0597 was computed in a linear regression between date and unique number of mutation per day.

Next, to obtain a result representative of the whole tree, we sampled at least 3 samples per date per haplotype and then completed the sampling up to a maximum of 1000 samples per haplotype. Sequences were put into a single fasta file, making sure we also have the presence of the Wuhan reference sequence representative (EPI_ISL_402119 representing EPI_ISL_402124, since the distance is null and the sampling date is older). The sites identified as problematic for phylogenetic tree reconstruction (flagged as "mask" in the problematic sites list v. 2021-04-15)[ref] were removed from the alignment using the relative reference genome positions on the multiple alignment.

The phylogenetic tree was then computed using FastTree v2.1.11 (55) using a GTR + Gamma model. The divergence tree was then refined using TreeTime (v. 0.7.4) (56). Without any corrections, the constructed phylogenetic tree using FastTree split haplotype I (Figure 2C), and combined very distinct lineages haplotype I and II. Thanks to our haplotype network, we were able to identify these connection issues and manually correct them. These inaccurate phylogenetic inner nodes are not unexpected given the instability shown by the local support values given by FastTree (<0.5). This can in part be overcome by removing noise in the alignment. By thinning the alignment using Gblocks, we obtained a more representative phylogenetic tree with the correct connections and successions of SARS-CoV-2 lineages during the first year of the pandemic (Figure S3). For this reason, we used the Gblocks thinned alignment to estimate mutation rate and TMRCA (Figure 2C). For completeness, we report that, without any thinning, we obtained a TMRCA in August 2019 and a mutational rate of 20.93 mutations/year.

To measure the mutational rate of each haplotype through the pandemic's first year we first used a root-to-tip molecular clock approach (Figure S4B) that was run on the divergence tree (Figure 2C). The root-to-tip distance was calculated using TempEst v1.5.3 (58) and tree visualization was made using ggtree (59). One sample was removed from the tree and the root-to-tip graph (EPI_ISL_833364) due to an incorrect sampling date (which was January 8 2020, prior to SARS-CoV-2 genome sequencing started), resulting in a total of 15,690 sequences. This root-to-tip molecular clock approach is slow and thus needs heavy down-sampling of sequences. Therefore, for the same sequences, we calculated the number of mutations from the reference and plotted them according to their date of sampling (Figure S4A). This latter approach is faster and takes advantage of all sequences available for a given time. Additionally, with this approach we can see some mutational "jumps" that are less apparent with the molecular clock. For example, in February, we saw some haplotype VII (also known as GISAID L lineage and Nextstrain 19B lineage) samples with a mutational jump (Figure S4A) that weren't as evident using the molecular clock (Figure S4B).

Details on Spurious Sites Flagging.

Upon manual inspection of consensus sequences, we noticed a systematic effect impacting regions of the genome around stretches of N for sequences from specific sequencing centres. We thus developed a script to evaluate this effect automatically. It identifies and removes spurious mutations resulting from this type of sequencing, as well as assembly errors.

Our script takes as input the FASTA file of sequence alignment (based on the reference genome) and the fasta file of the reference sequence. The sequences in the alignment must be exactly the length of the reference genome (here, 29 903 nucleotides). Two parameters can be specified: the first parameter (alpha) corresponds to the minimum number of consecutive unknown nucleotides (N) in a stretch (see Figure 2); the second parameter (beta) corresponds to the maximum distance, in nucleotides, between a putative variant and the stretch of Ns. Here, we used alpha=5 and beta=10.

In a specific sequence from the FASTA alignment file, the script will find all stretches of at least alpha consecutive Ns. Each nucleotide within beta positions of every stretch of N will be compared to the nucleotide at the corresponding position in the reference genome: a position which differs will be identified as a putative spurious mutation (flagged) and replaced with an unknown nucleotide in the output fasta file. It is also possible to specify a bed file of positions to investigate. In this case, the script won't look at all positions, but rather will only flag positions specified in the bed file. This option speeds up processing if the user is only interested in a specific region of the genome. We also added an option to protect specific positions from being flagged, such as known real mutated positions that could inadvertently be located next to stretches of Ns. The user can give a bed file with or without a column containing the alternative allele at the protected position. If there is no alternative allele specified, the position will not be considered spurious (but will be reported). If an allele is specified, the script will consider it spurious only if the nucleotide at that position differs from the reference and from the specified alternative allele.

The output the script consists of three files: a corrected alignment file in FASTA format where flagged positions are replaced by Ns; a file reporting the number of flagged sites per sequence; and a file with information on every flagged site: sequence ID, position, the distance from the closest stretch of Ns, the flag, and the nucleotide found at this position. The flag is set to 0 when the site is spurious, set to 1 if the site is protected, or set to 2 if the site is protected but differs from the alternative allele reported. Only positions flagged with 0 and 2 are replaced by N in the output FASTA file.

Time-dependent Haplotype Network Optimization

For each consensus sequence, the sampling date was rounded into half-month date intervals. Next, each haplotype was associated to a date interval by identifying its first occurrence. For each interval, a network was generated using pegas (54) with only haplotypes that occurred before or within the interval. The networks were then merged iteratively over time. To avoid circular connections between haplotypes during the merging process, the circular patterns in the network were removed by first identifying these patterns, then removing the links between networks with the longest time interval difference. However, if the merging created a cycle, we only kept the branches of a cycle that linked the haplotypes with the lowest time difference.

Supplementary Tables

Table S1. Mutation Frequency Spectrum
Number of mutated genomic positions within the GISAID sequences binned by the whole year, the first, or the second wave of the pandemic. The rows represent the mutated stratified by their appear in each group of sequences.

Number of consensus sequences with mutation	Total 2020 mutation counts	First Wave mutation counts	Second Wave mutation counts
1	3,168	4,758	3,695
2 - 10	9,877	9,776	9,471
11 - 99	8,622	4,862	6,646
>100	3,135	1,007	2,398
total	24,802	20,403	22,210

Table S2. The 22 mutated genomic positions that represent the genetic diversity of SARS-CoV-2 during the first year of the pandemic.
These 22 positions define the haplotype groupings. For each mutetd position, the frequencies per wave and for the whole year are also represented. Additionally, for each position, we report their substitution, gene position, amino acid change, and functional consequence on the resulting protein.

Genomic position	Frequency 2020	Frequency first wave	Frequency second wave	Nuc acide Substitution	Gene	Amino acide Subsitutition	Functional consequence
**241**	94%	88%	100%	C>T	intergenic	N/A	N/A
**313**	4%	7%	1%	C>T	nsp1	L16L	synonymous
**1059**	14%	21%	8%	C>T	nsp2	T265I	missense
**1163**	5%	7%	2%	A>T	nsp2	I300F	missense
**3037**	94%	88%	100%	C>T	nsp3	F924F	synonymous
**7540**	3%	6%	0%	T>C	nsp3	T2425T	synonymous
**8782**	2%	5%	0%	C>T	nsp4	S2839S	synonymous
**14408**	94%	88%	100%	C>T	nsp12	P4720L	missense
**14805**	3%	4%	2%	C>T	nsp12	Y4852Y	synonymous
**16647**	3%	6%	1%	G>T	nsp13	T5466T	synonymous
**18555**	3%	6%	1%	C>T	nsp14	D6102D	synonymous
**22227**	23%	1%	44%	C>T	S	A222V	missense
**22992**	6%	6%	6%	G>A	S	S477N	missense
**24334**	5%	0%	9%	C>T	S	A924A	synonymous
**23401**	3%	6%	0%	G>A	S	Q613H	missense
**23403**	94%	88%	100%	A>G	S	D614G	missense
**25563**	22%	27%	17%	G>T	ORF3a	Q57H	missense
**26144**	2%	3%	0%	G>T	ORF3a	G251V	missense
**28144**	2%	5%	0%	T>C	ORF8	L84S	missense
**28881**	34%	41%	28%	G>A	N/orf9c	N:R203K	missense
**28882**	34%	41%	27%	G>A		N:G204R	
**28883**	34%	41%	27%	G>C		/ORF9c:G50N	

Supplementary Figures

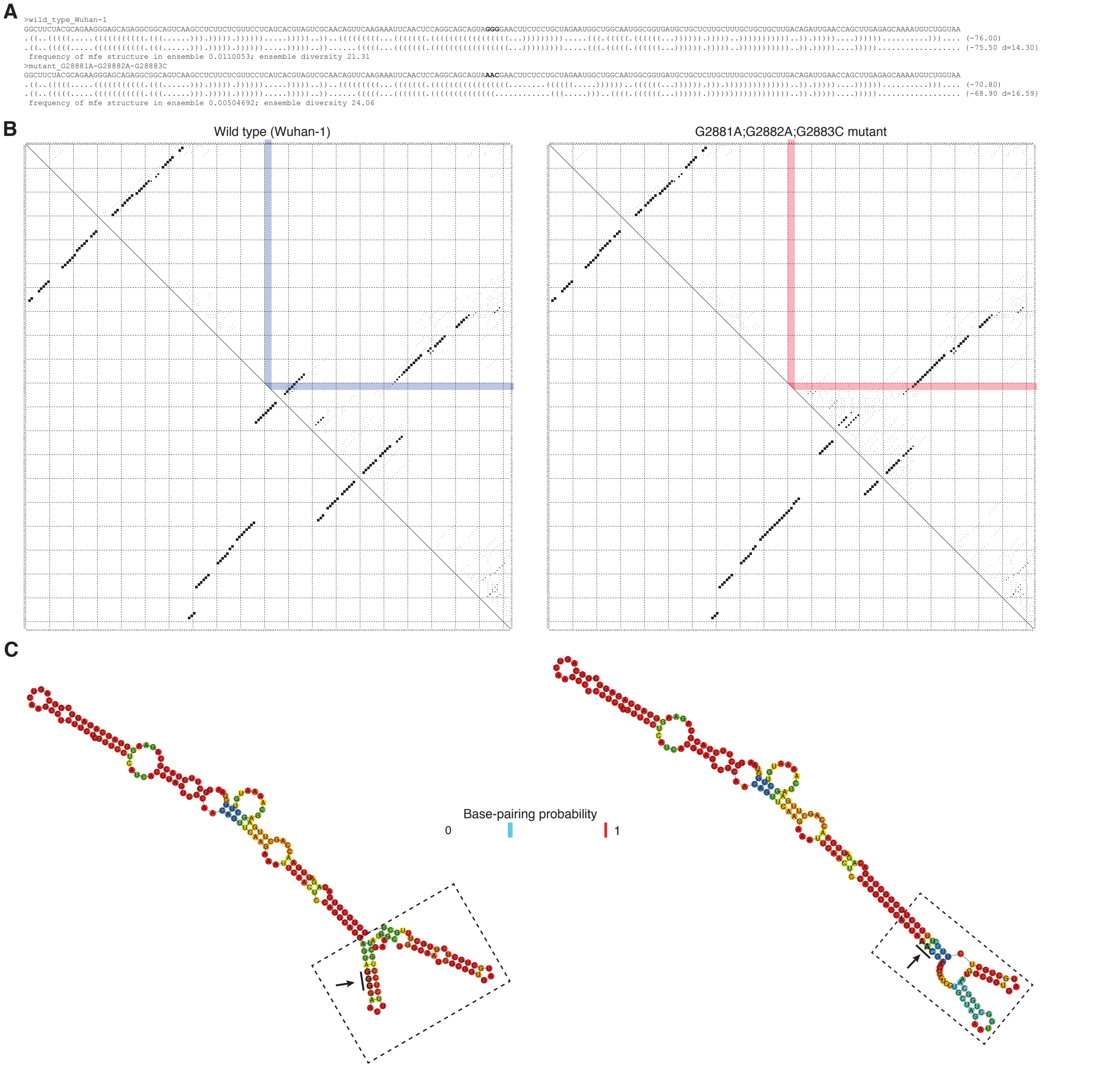

Fig. S1: **Comparative secondary structure prediction of reference SARS-CoV-2 transcript *vs* trinucleotide ORF9c mutant.**

(A) Primary sequence, minimum free energy (MFE) RNA secondary structure prediction (RNAfold with partition function, ViennaRNA package) and centroid structure prediction with MFE frequency in – and diversity of – Boltzmann ensemble. (B) Base-pair probability matrices for reference sequence (left) and trinucleotide mutant sequence (right) with the position of affected nucleotides highlighted in colour. The size of dots reflects the base pairing probability distribution in the Boltzmann ensemble of suboptimal base pairings (right of diagonal), whilst the MFE prediction is illustrated left of the diagonal. (C) RNA secondary structure predictions annotated with base-pairing probabilities. Dotted boxes highlight the topological differences associated with the trinucleotide mutation (arrow).

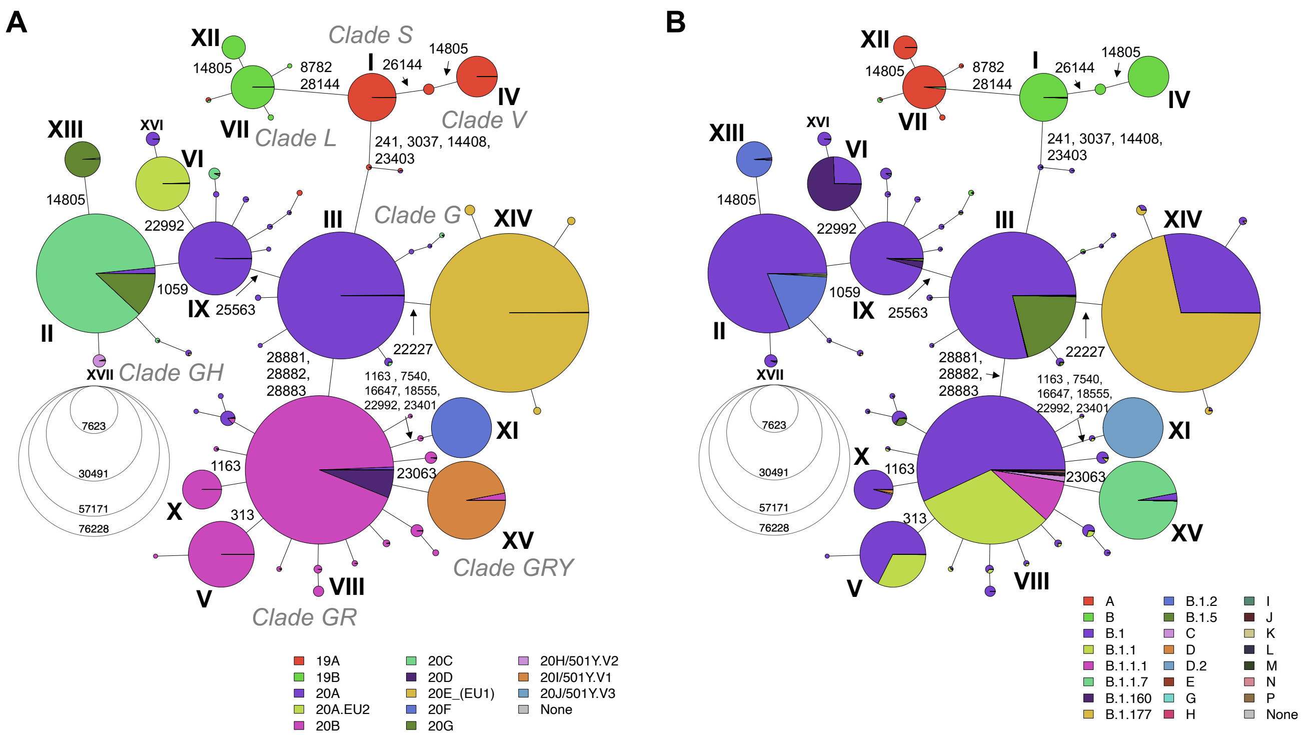

Fig. S2: **Haplotype Networks with NextStrain and Pangolin lineage correspondence.**
(A) Haplotype Network with NextStrain correspondence, and GISAID clade annotation labeled in grey outside the pie charts. Each branch represents the mutation that define the haplotype lineage. (B) Haplotype Network with Pangolin lineage correspondence. The pangolin lineage has a colour only if there are more than 5000 samples with this lineage. We added those with at most 5000 to the sub-lineage (the letter+one number), and if even this sub-lineage is no more than 5000, we only kept the first letter.

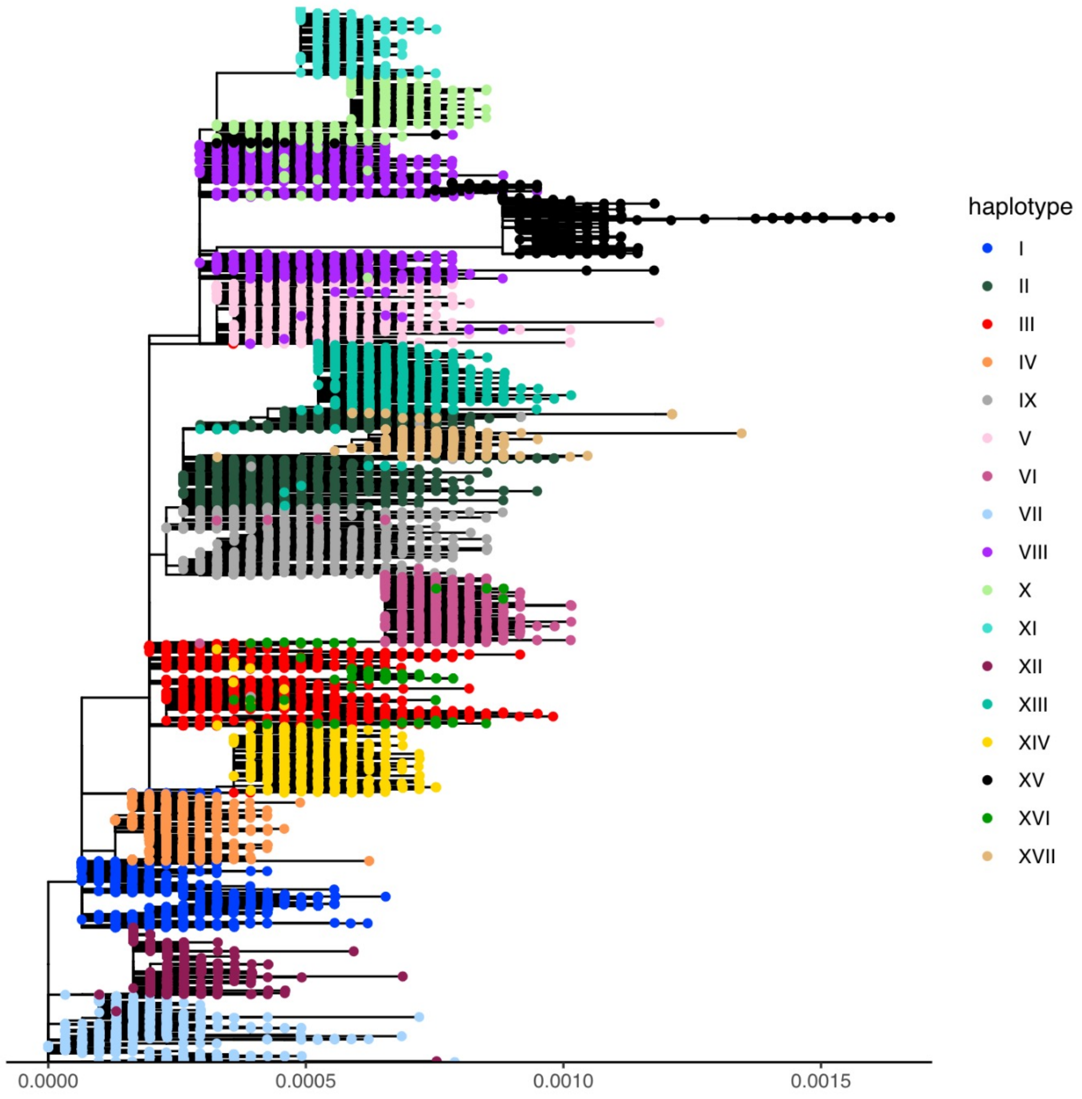

Fig. S3: **Unrooted phylogenetic tree summarizing the circulating lineages during the first year of the pandemic.** The phylogenetic tree was built using the same samples as what is seen in Figure 2C, the alignment was post-treated using the program Gblocks (34), which eliminates poorly aligned positions and divergent regions of a multiple sequence alignment. Default parameters were used. The tree was then built using FastTree using a GTR+Gamma20 model and TreeTime to refine the divergence tree.

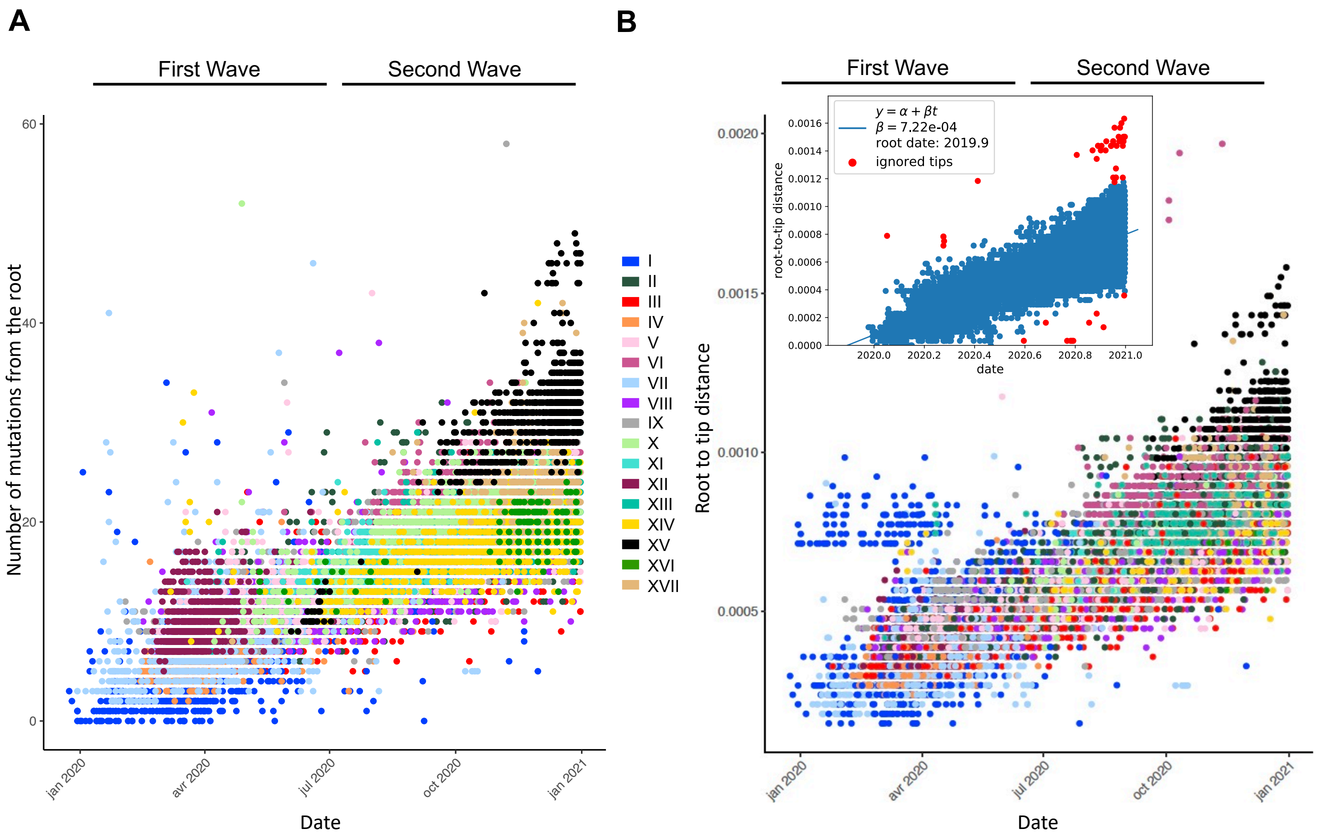

Fig. S4: **Different approaches to measure mutational jumps in lineages**
(A) Number of mutations compared to the reference genome (NC_045512.2) for the same samples as the ones in Figure 2C and coloured using the haplotype annotation.(B) Root-to-tip distance plot based on the phylogenetic divergence tree build using sub-sampled GISAID SARS-CoV-2 consensus sequences, which are coloured using the haplotype annotation. The root-to-tip distance is measured from the phylogenetic tree presented in Figure 2C using TempEst v.1.5.3 (58). GBlocks was used for thinning of the TimeTree top left to compute the time to a most recent common ancestor (TMRCA) and the mutation rate. The TMRCA date is 2019.9 representing October 2019 ((0.9 of year 2019), and the mutation rate which represents the nucleotide changes per position for the year is 7.22e-4. This mutation rate translates to 21.60 mutations/year.

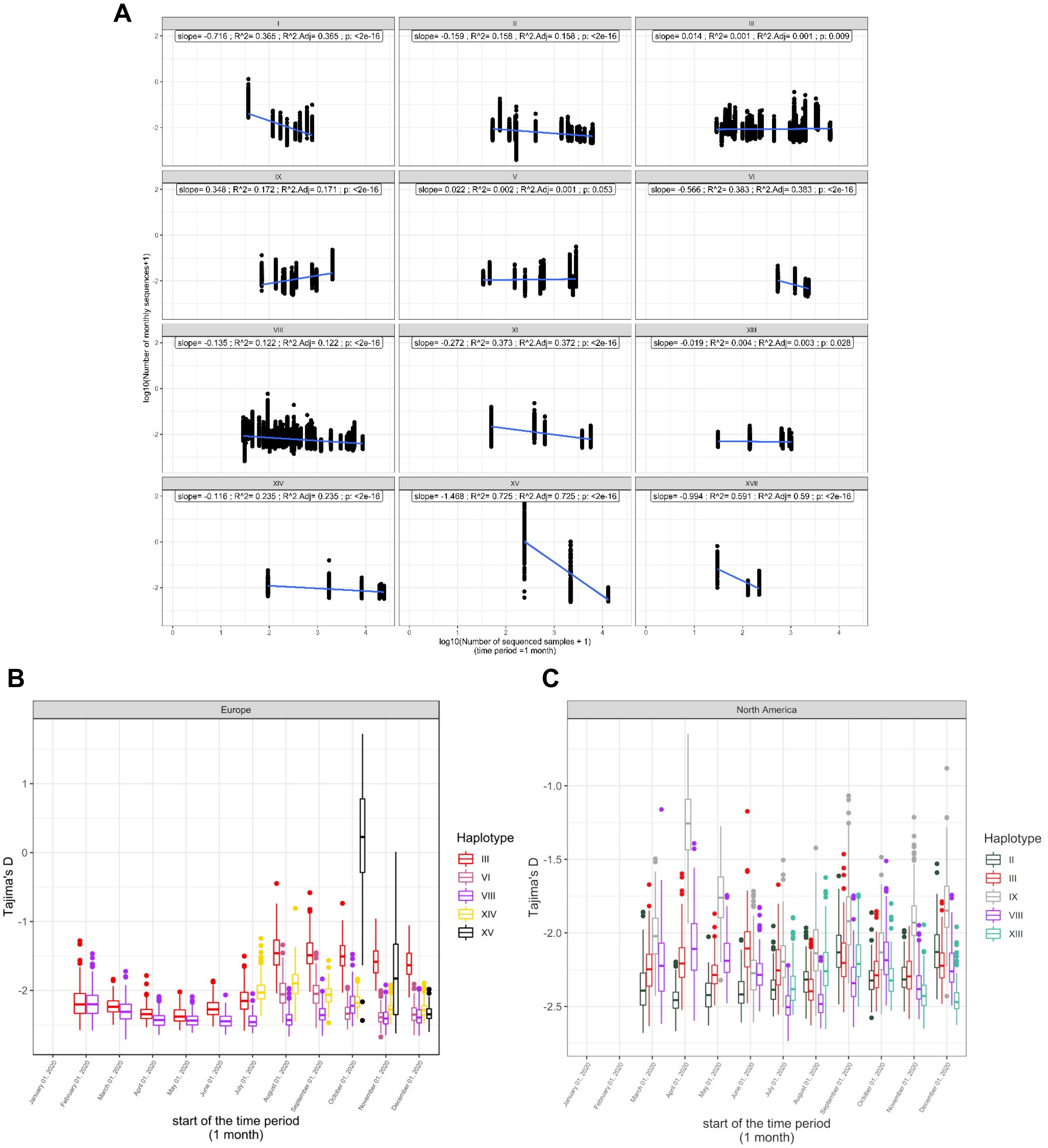

Fig. S5: **Tajima's D correlation with haplotype trajectories over time in each continent**
(A) R^2 of Tajima's D across haplotypes. For most of the haplotypes, Tajima's D correlates negatively and exponentially with the number of sequences per haplotype per continent per month. This value is used as a proxy for the number of cases per haplotype per month per continent. For almost all the haplotypes (I, II, III, IX, VI, VIII, XI, XIII, XIV, XV, and XVII), the correlation is significant (PERMANOVA $p < 0.05$) with a mean adjusted r^2 of 0.24 across haplotypes (s.d. = 0.23). This correlation is consistent with the fact that smaller negative Tajima's D values are associated with stronger spread and that Tajima's D can be used for phylodynamic inferences. The moderate correlation strength (r^2) can be explained by the fact that the number of sequences is probably an underestimate of the number of true cases and by the fact that the analysis captures the average trend in each continent, which is weakened by variations of epidemiological dynamics across countries and cities. Tajima's D estimates of the five most prevalent haplotypes in Europe (B) and North America (C) for the first year of the pandemic. Boxplots represent 500 estimates of Tajima's D from random resamplings of 20 genome sequences for each month with at least 20 sequences.

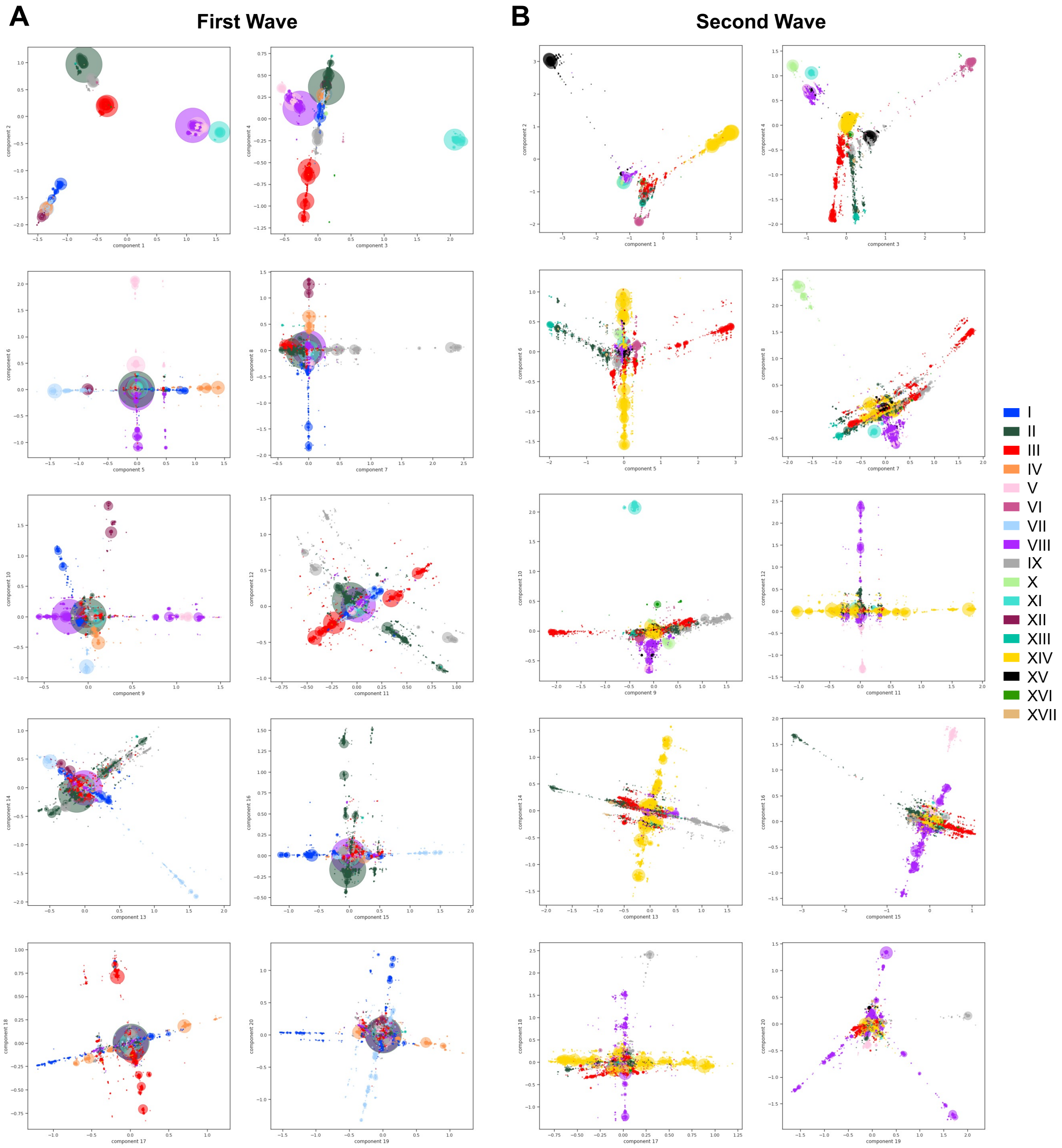

Fig. S6: **Viral population structure during the first and second waves of the pandemic across all 20 Principal Components (PCs)**.
Principal Component Analyses (PCA) of genetic diversity of the first (A) and second (B) waves' consensus sequences shown for the 20 first PCs. Genetic variation present in at least 10 genomes is used. The PCA was computed with all sequences, and only the sequences from the 17 main haplotypes are projected. Identical sequences are projected onto the same coordinates, therefore the number of sequences represented by each point is proportional to the size of the dots, with added transparency.

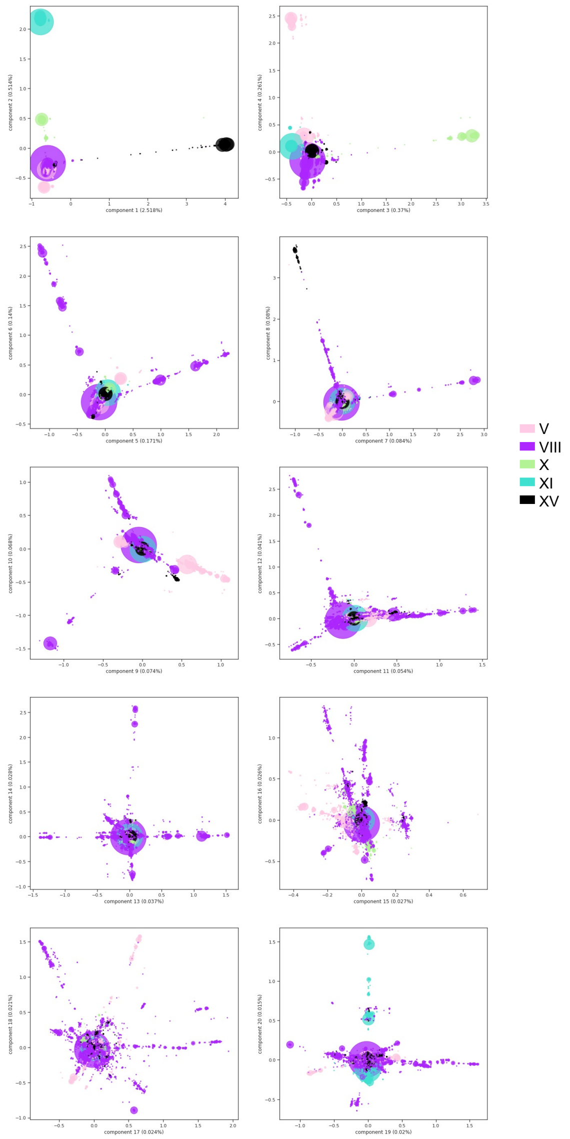

Fig. S7: **20 PCs derived from samples annotated haplotype VIII and descendants**
Principal Component Analyses (PCA) of genetic diversity of haplotype VIII and its descendants (haplotypes V, X, XI, and XV) annotated GISAID sequences shown for the first 20 PCs. Positions mutated in at least 10 sequences within this subgroup were used. Identical sequences are projected onto the same coordinates, and the number of sequences that each coordinate represents is proportional to the size of the dot.

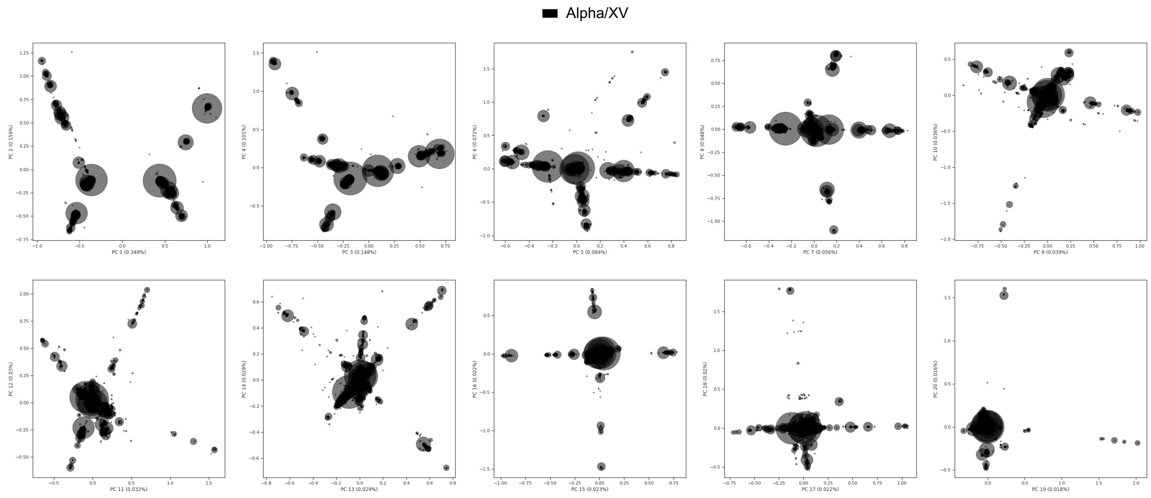

Fig. S8: **20 PCs derived from samples annotated Alpha by the Pangolin lineage annotation**
Principal Component Analyses (PCA) of genetic diversity of Alpha annotated sequences by Pangolin on January 19th 2021, and which are part of haplotype XV. Positions mutated in at least 10 sequences within this subgroup were used. Identical sequences are projected onto the same coordinates, and the number of sequences that each coordinate represents is proportional to the size of the dot.

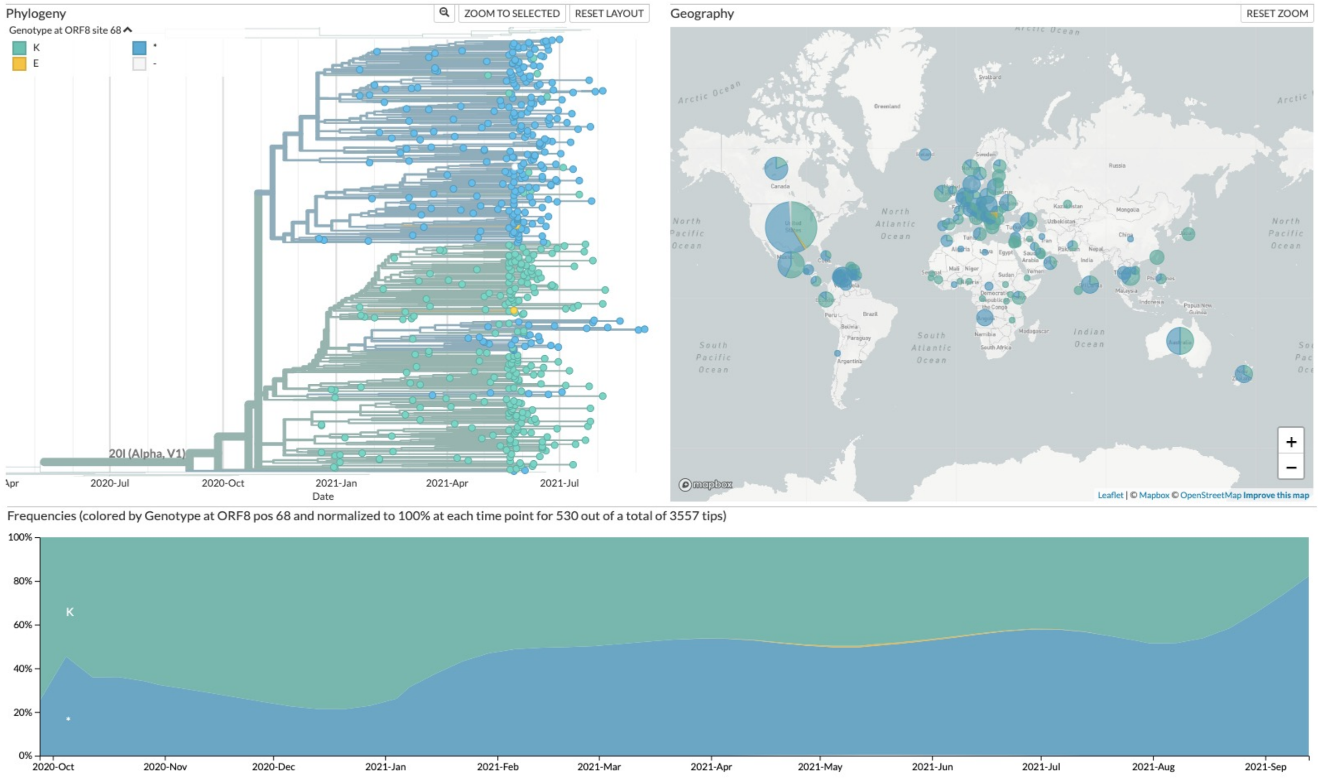

Fig. S9: **NextStrain frequencies of mutation A29095U (ORF8:K68*) on the Alpha variant identified by a using PCA**
NextStain phylgentic tree, world-wide frequency, and time-series frequency of samples with mutation ORF8:K68*. Figures captured from nextstrain.org/sars-cov-2 on September 29th 2021.
